# Genomic characterization of equine influenza A subtype H3N8 viruses by long read sequencing and functional analyses of the PB1-F2 virulence factor of A/equine/Paris/1/2018

**DOI:** 10.1186/s13567-024-01289-8

**Published:** 2024-03-22

**Authors:** Lena Kleij, Elise Bruder, Dorothée Raoux-Barbot, Nathalie Lejal, Quentin Nevers, Charlotte Deloizy, Bruno Da Costa, Loïc Legrand, Eric Barrey, Alexandre Chenal, Stéphane Pronost, Bernard Delmas, Sophie Dhorne-Pollet

**Affiliations:** 1 Unité de Virologie et Immunologie Moléculaires, INRAE, UVSQ, Université Paris-Saclay, 78350 Jouy-en-Josas, France; 2 CNRS UMR 3528, Biochemistry of Macromolecular Interactions Unit, Department of Structural Biology and Chemistry, Institut Pasteur, Université Paris Cité, 75015 Paris, France; 3 LABÉO Frank Duncombe, 14280 Saint-Contest, France; 4 BIOTARGEN, Normandie Univ, UNICAEN, 14000 Caen, France; 5 AgroParisTech, Unité de Génétique Animale et Biologie Intégrative, INRAE, Université Paris-Saclay, 78350 Jouy-en-Josas, France

**Keywords:** Nucleotide sequencing, equine influenza, equine influenza virus, H3N8, nanopore, PB1-F2, virulence, long-read sequencing, virus, horse

## Abstract

Equine influenza virus (EIV) remains a threat to horses, despite the availability of vaccines. Strategies to monitor the virus and prevent potential vaccine failure revolve around serological assays, RT-qPCR amplification, and sequencing the viral hemagglutinin (HA) and neuraminidase (NA) genes. These approaches overlook the contribution of other viral proteins in driving virulence. This study assesses the potential of long-read nanopore sequencing for fast and precise sequencing of circulating equine influenza viruses. Therefore, two French Florida Clade 1 strains, including the one circulating in winter 2018–2019 exhibiting more pronounced pathogenicity than usual, as well as the two currently OIE-recommended vaccine strains, were sequenced. Our results demonstrated the reliability of this sequencing method in generating accurate sequences. Sequence analysis of HA revealed a subtle antigenic drift in the French EIV strains, with specific substitutions, such as T163I in A/equine/Paris/1/2018 and the N188T mutation in post-2015 strains; both substitutions were in antigenic site B. Antigenic site E exhibited modifications in post-2018 strains, with the N63D substitution. Segment 2 sequencing also revealed that the A/equine/Paris/1/2018 strain encodes a longer variant of the PB1-F2 protein when compared to other Florida clade 1 strains (90 amino acids long versus 81 amino acids long). Further biological and biochemistry assays demonstrated that this PB1-F2 variant has enhanced abilities to abolish the mitochondrial membrane potential ΔΨm and permeabilize synthetic membranes. Altogether, our results highlight the interest in rapidly characterizing the complete genome of circulating strains with next-generation sequencing technologies to adapt vaccines and identify specific virulence markers of EIV.

**Supplementary Information:**

The online version contains supplementary material available at 10.1186/s13567-024-01289-8.

## Introduction

Equine influenza (EI) is a highly contagious respiratory disease affecting horses, with significant economic repercussions on the global equine industry [1–4]. Its widespread transmission is facilitated by the international transport of horses, primarily for competition and breeding purposes [5, 6]. Common clinical manifestations of EI infection in naïve and unprotected animals include pyrexia, persistent cough, serous nasal discharge, dyspnea, muscle pain or weakness, lethargy, anorexia, and often complications arising from secondary bacterial infections [7, 8]. Although rarely fatal on its own, EI can lead to secondary bacterial infections in the respiratory tract and lungs, exacerbating the clinical condition of affected horses [4, 8].

Equine influenza virus (EIV), which is the causal agent of EI, is an influenza type A virus belonging to the *Orthomyxovirus* genus within the *Orthomyxoviridae* family. Currently, EI is known to be caused by only two primary virus subtypes: H3N8 and H7N7, with the latter remaining undetected since the 1970s [9]. The H3N8 subtype emerged in 1963 [10] in the Americas and has since spread globally, continuing to trigger epizootic events [2, 3, 11–13]. In the 1980s, H3N8 further diverged into American and Eurasian lineages [14]. The American lineage subsequently branched into the Kentucky, South American, and Florida sublineages [15]. The Florida sublineage underwent additional evolution in the early 2000s, resulting in two subtypes: Florida sublineage clade 1 (FC1) and Florida sublineage clade 2 (FC2) [16]. FC1 predominantly circulated in the Americas, while FC2 prevailed in Europe. However, this pattern shifted with the 2009 outbreak of an FC1 strain in Europe [17, 18]. Subsequently, EIV FC1 caused an outbreak of an unprecedented scale between late 2018 and 2019 in Europe [12, 19], with 53 outbreaks reported in France, 228 in the United Kingdom, and approximately 80 in Ireland [20, 21]. During the 2018 outbreak, vaccination coverage was substantial in France [20]. The vaccines used during these outbreaks are still considered effective by the World Organization for Animal Health Expert Surveillance Panel (OIE ESP) [20–22].

Currently, most diagnostic tests for EIV rely on detecting viral antigens or RT-qPCR amplification of viral nucleic acids obtained from nasal swab samples. These two approaches have distinct trade-offs: antigen testing is swift but has limited sensitivity, while RT-qPCR is more time-consuming but offers higher sensitivity. Moreover, data generated by these methods have limitations in providing insights into epidemiological links and vaccine effectiveness. In most cases, sequencing of the viral strains is performed posteriorly by Sanger sequencing using several segment-specific primers [23]. This technique is efficient but very time-consuming, and multiplexing is not possible. Therefore, there is a need to develop new diagnostic tools that combine speed, sensitivity, ability to detect coinfections, and comprehensive genome sequence information. Such methods are vital for effective health management strategies, including the identification of potential new virulence factors and the precise design of vaccines.

In this study, our objective was to genetically characterize the equine influenza H3N8 viruses circulating in France during the winters of 2009 and 2018 and, more specifically, to identify and characterize potential virulence determinants and antigenicity through whole-genome sequencing. Therefore, we used MinION long-read sequencing technology, which offers rapid sequencing and multiplex barcoding [24–27]. The viral strains A/equine/Beuvron-en-Auge/2/2009 and A/equine/Paris/1/2018, along with the OIE-recommended vaccine strains A/equine/Richmond/1/2007 and A/equine/South Africa/4/2003, were sequenced. Our results suggest that the accessory protein PB1-F2 previously characterized as a virulence factor in mammals [28] may contribute to the virulence of the A/equine/Paris/1/2018 strain.

## Materials and methods

### Cell cultures

A549 cells (human alveolar epithelial cells, American Type Culture Collection) and MDCK cells (Madin-Darby Canine Kidney cells, ATCC) were cultured in minimal essential medium (MEM) (Merck) containing 2 mM l-glutamine, 100 IU/mL penicillin, 100 μg mL^−1^ streptomycin, and 10% fetal bovine serum. Cells were maintained at 37 °C in a 5% CO_2_ incubator.

### Viruses

Equine influenza viruses (EIV) H3N8 A/equine/Beuvron-en-Auge/2/2009 [11], A/equine/Paris/1/2018 [20], and the vaccine strains [16] A/equine/Richmond/1/2007 and A/equine/South Africa/4/2003 were isolated from sick horses during respiratory disease outbreaks. The nasopharyngeal swabs collected were placed in 5 mL of virus transport medium containing minimum essential medium supplemented with 10% fetal bovine serum and 1% w/v antibiotics (penicillin, streptomycin, and amphotericin). All the EIV viruses used in this study were first amplified by passaging in 11-day-old embryonated chicken eggs (PA12 White Leghorn strain). Inocula were injected into the allantoid cavity (100 µL per egg). A second virus amplification step was carried out in 25 cm^2^ flasks of MDCK cell monolayers. When cell lysis was observed, cultures were stopped, and RNA extraction was performed immediately.

### RNA extraction

Extraction of EIV RNA from EIV-infected MDCK cells was carried out using TRIzol LS Reagent (Life Technologies) and further purified using the RNeasy MinElute clean-up kit (Qiagen) according to the manufacturer’s recommendations. RNA integrity was assessed on an Agilent 2100 Bioanalyzer using the RNA 6000 nano kit (Agilent, Santa Clara, CA, USA) following the manufacturer’s instructions. We monitored RNA yield and purity with a NanoDrop ND-2000c spectrophotometer.

### MinION long-read library preparation, sequencing and data analysis

#### cDNA synthesis

Purified RNA was reverse transcribed using SuperScript III (Thermo Scientific) and primers designed by [29] and complementary to the conserved 3ʹ end of influenza A vRNA. We used primers RTA-U12 (5ʹ-AGCAAAAGCAGG) expected to target the segments PA, NP, M, NS and RTA-U12.4 (5ʹ-AGCGAAAGCAGG) expected to target the segments PB2, PB1, HA, NA, combined in a 2:3 molar ratio [29]. 500 ng of total RNA and 10 pmol of specific primers (2:3 molar ratio RTA-U12, RTA-12.4) were denatured for 5 min at 65 °C, centrifuged, and stored on ice before adding the reaction mix, according to the manufacturer’s instructions. We incubated the RT reactions at 25 °C for 10 min and then 50 °C for 60 min. The reaction was then stopped by heating at 70 °C for 15 min. After cDNA synthesis, RNA was degraded by incubation with 2 U of RNase H for 20 min at 37 °C. The RNA hydrolysis reaction was stopped by heating at 70 °C for 10 min, and the cDNAs were stored at −20 °C until use. We evaluated the quantity and quality of cDNA on sixfold dilutions with the RNA 6000 Pico kit (Agilent) on an Agilent 2100 Bioanalyzer.

#### cDNA amplification

The eight influenza A genomic segments were amplified by PCR using the cDNA previously produced. Platinum II Taq Hot start DNA-polymerase (Invitrogen) was used according to the manufacturer’s instructions, with primers set complementary to the 5ʹ and 3ʹ ends of each influenza A genome segment (Additional file 1). Amplified DNA products were purified using AMPure XP beads (Beckman Coulter Inc., Pasadena, CA, USA) at a ratio of 1.2:1 volume of beads per sample, and DNA yield was monitored with a NanoDrop ND-2000c spectrophotometer and a Qubit fluorimeter using a Qubit dsDNA BR kit (Invitrogen).

#### Nanopore sequencing and data analysis

For each of the four strains, the eight purified PCR products were pooled at an equimolar ratio and used as input for library generation using the Ligation Sequencing Kit SQK-LSK109 and the Native Barcoding Expansion 1–12 kit EXP-NBD104 according to the manufacturer’s instructions (Oxford Nanopore Technologies). The barcode-ligated DNA samples were pooled at an equimolar ratio and used for final adapter ligation. We loaded 50 fmol of the purified adapter-ligated DNA library onto a MinION Flow-cell (R9.4.1; FLO-MIN106D) and run it on a MinION Mk1C device according to the manufacturer’s instructions. Guppy (version 5.1.13) was used for basecalling and demultiplexing. Nanofilt (version 2.8.0) was used to filter reads based on their size and quality: size > 600 bp, Q > 10. The filtered reads were mapped using minimap2 (version 2.22) and A/equine/Ohio/113461-1/2005 as the reference genome (GenBank accession numbers: CY067323, CY067324, CY067325, CY067326, CY067327, CY067328, CY067329, CY067330). SAMtools (version 1.14) was used to convert the data into bam and medaka (version 1.4.4) for variant calling. Finally, the Integrative Genomics viewer desktop application (IGV, version 2.16.2) was used for visualization.

The newly sequenced viral genomes have been deposited in the European Nucleotide Archive under project accession numbers GCA_963870925 for A/equine/Paris/1/2018, GCA_963870635 for A/equine/Beuvron-en-Auge/2/2009, GCA_963870835 for A/equine/Richmond/2007 and GCA_963870825 for A/equine/South Africa/4/2003.

### Sequence multialignment and phylogenetic trees

A multiple alignment of all nucleotide sequences of the eight genes of equine influenza of type A H3N8 was obtained using Muscle. Evolutionary analyses were conducted in MEGA11 [30, 31] using the maximum likelihood method and the Hasegawa-Kishino-Yano substitution model [32]. The tree with the highest likelihood is shown. The percentage of replicate trees in which the associated taxa clustered together in the bootstrap test 1000 replicates [33] are shown next to the branches. Initial tree(s) for the heuristic search were obtained automatically by applying neighbor-joining and BioNJ algorithms to a matrix of pairwise distances estimated using the maximum composite likelihood (MCL) approach and then selecting the topology with superior log likelihood value. A discrete Gamma distribution was used to model evolutionary rate differences among sites [5 categories (+ G, parameter)]. The codon positions included were 1st + 2nd + 3rd + Noncoding [30, 31]. All accession numbers are listed in Additional file 2.

The amino acid sequences of viral proteins (PB2, PB1, PB1-F2, PA, PA-X, HA, NP, NA, M1, M2, NS1, and NEP) from recent EIV isolated in France were aligned with the strain A/equine/Ohio/1/2005 used as a reference for consensus sequence construction using Clustal Omega from EMBL-EBI [34] and Unipro UGENE [35].

### Plasmids

Codon-optimized open reading frames encoding HA-tagged versions of PB1-F2 of viral strains A/equine/Ohio/1/2003 and A/equine/Paris/1/2018 were cloned in the eukaryotic expression vector pCAGGS at the Not I and Bgl II restriction sites. Codon-optimized open reading frames encoding His-tagged versions of PB1-F2 of A/equine/Ohio/1/2003 and A/equine/Paris/1/2018 were cloned in the bacterial expression vector pET-28a+ at the Nde I and Xho I restriction sites.

### Immunohistochemistry—confocal microscopy

A549 cells were seeded at 0.5 × 10^6^ cells per well on 18 mm diameter glass lamellas and incubated for 24 h at 37 °C and 5% CO_2_. Cells at 80–90% confluence were transfected with 200 ng of pCAGGS derivates using Lipofectamine® 2000 (11668027, Thermo Fisher Scientific) following the manufacturer’s instructions. Forty hours post-transfection, MitoTracker CMX Ros (M7512, Thermo Fisher Scientific) was added to the cell culture at a final concentration of 500 nM for 30 min. Next, after cell culture medium removal, the cells were fixed using 4% paraformaldehyde for 30 min at room temperature (RT). Cell monolayers were washed in phosphate saline buffer (PBS) and PBS completed with 0.1% Triton X-100 (PBS-Tx) and with 1% w/v bovine serum albumin (BSA) for 1 h at RT. The cells were then incubated with a rabbit anti-HA-tag antibody (H6908, Sigma‒Aldrich) in PBS-Tx supplemented with 0.2% BSA. After three washes in PBS-Tx, an anti-rabbit immunoglobulin goat antibody labeled with Alexa Fluor 488 (A11008, Invitrogen, OR, USA) in PBS-Tx completed with 0.2% BSA was added for 2 h at RT. Nuclei were marked with Hoechst diluted to 1/100 in PBS 1× for 5 min at RT. Subcellular localization images were taken using a Zeiss LSM 700 confocal 187 microscope with a ×63 objective.

### PB1-F2 production in *E. coli* and purification

BL-21 Rosetta cells (Stratagene) were transformed with the resulting plasmids and cultured to an optical density (OD) of 0.8 before overnight incubation at 28 °C in 1 mM isopropyl 1-thio-β-D-galactopyranoside (IPTG) under agitation. Next, bacteria were pelleted and resuspended in 50 mM Tris (pH 7.4), 10 mM EDTA, and 0.1% Triton X-100 buffer and incubated at 37 °C for 30 min. The suspension was sonicated and centrifuged at 10 000 × *g* for 30 min at 4 °C. Pellets were resuspended in solubilization buffer (20 mM Tris (pH 7.4), 0.5 M NaCl, 5 mM imidazole, and 8 M urea) and centrifuged at 10 000 × *g* for 30 min at 4 °C. Supernatants were sonicated and filtrated using 0.8 µm filters (SLAAR33S, MilliporeSigma™ Millex™) before loading on a Histrap FF IMAC column (17531901, Cytiva) using the AKTA Purifier 100 FPLC chromatographic system (GE Healthcare). Fractions containing PB1-F2 were pooled and subjected to size exclusion chromatography on a Sepharose S200 column equilibrated with solubilization buffer. Next, urea was removed from the S200 PB1-F2-containing fractions on a 53 mL HiPrep™ 26/10 Sephadex G-25 resin column (GE17-5087-01, Sigma‒Aldrich) equilibrated with 5 mM ammonium acetate buffer, pH 5. Fractions containing PB1-F2 were lyophilized and stored at −20 °C. Prior to their use, lyophilized PB1-F2 powder was dissolved in 5 mM sodium acetate buffer (pH 5). Protein concentration was estimated by measuring OD at 280 nm and using the extinction coefficients of 23,490 M^−1^ cm^−1^ for the Ohio protein and 37,470 M^−1^ cm^−1^ for its Paris homolog.

### Lipid vesicle preparation

(16:0–18:1) 1-Palmitoyl-2-oleoyl-sn-glycero-3-phospho-l-serine (POPS) (840034), 1-palmitoyl-2-oleoyl-sn-glycero-3-phospho-(1ʹ-rac-glycerol) (PG) (840457), and (18:1) cardiolipin 1ʹ,3ʹ-bis[1,2-dioleoyl-sn-glycero-3-phospho]-glycerol (DOCL) (840044) were purchased from Avanti Polar Lipids (Alabaster, AL, USA). (16:0–18:1) 1-Palmitoyl-2-oleoyl-glycero-3-phosphocholine (POPC) (37-1618-9), (16:0–18:1) 1-palmitoyl-2-oleoyl-sn-glycero-3-phosphoethanolamine (POPE) (37-1828-7), and soybean l-α-phosphatidylinositol (PI) (37-0130-7) were purchased from Larodan (France). ANTS (FP-46574B, 8-aminonapthalene-1,3,6 trisulfonic acid) and DPX (FP-47017A, p-xylene-bis-pyridinium bromide) were purchased from Interchim (Montluçon, France). Sodium acetate buffers and phosphate buffers were of analytical grade. Reagents for SDS‒PAGE electrophoresis were obtained from Invitrogen (France).

Lipids POPC, POPE, POPS, PI, and DOCL were used at a molar ratio of 5.5:2.5:1.5:1:0.5 to mimic mitochondria outer membranes (OMM). The mix of lipids with 20 mM ANTS (fluorophore probe) and 60 mM DPX (quencher) in a final concentration of 10 mM sodium acetate (pH 5) was sonicated using a sonicator tip to obtain an emulsion. Reversed-phase evaporation was carried out using a Heidolph Laborota 4003 apparatus to obtain large unilamellar vesicles (LUVs). LUV preparations were extruded three times through a Swinny filter (XX3001200, Millipore) using polycarbonate filters with pore size diameters of 1.2 μm, 0.4 μm and 0.2 μm (Merck Millipore, Darmstadt, Germany). Unencapsulated ANTS and DPX were removed by gel filtration through a 5 mL HiTrap Desalting Sephadex G-25 resin column (GE Healthcare Life Sciences). To ensure the correct size and obtain LUVs, dynamic light scattering (DLS) measurements were performed on a Nano series Zetasizer (Malvern Instruments, Paris, France).

### Lipid vesicle permeabilization assay

For permeabilization assays, LUVs were incubated at 0.4 mM lipid concentration in 10 mM sodium acetate (pH 5) at 25 °C in a black p96-well plaque (Greiner), and fluorescence titrations were performed with an FP-8200 Jasco spectrofluorometer equipped with a Peltier-thermostated ETC-272T (25 °C). The excitation wavelength was set at 360 nm, and the emission of ANTS was measured between 500–600 nm at a bandwidth of 5 nm to ensure that the signal perceived was indeed permeabilization and not unspecific diffraction. The intensity was measured before and after the addition of PB1-F2 at final concentrations of 1 μM, 500 nM, 250 nM, 100 nM, and 50 nM. The maximum intensity of permeabilization, corresponding to the maximum intensity of ANTS fluorescence, was measured after the addition of 0.1% (v/v) Triton X-100. The experiment was carried out 4 times in triplicate. Statistical analysis was carried out with REML F(1,99) = 55.01, *P* < 0.0001 and Šídák’s multiple comparison (1 μM *P* value = 0.0021; 500 nM *P* value = 0.0011; 250 nM *P* value = 0.0003) on Prism v9.

## Results

### Full-length genome sequencing strategy

**Figure 1:**
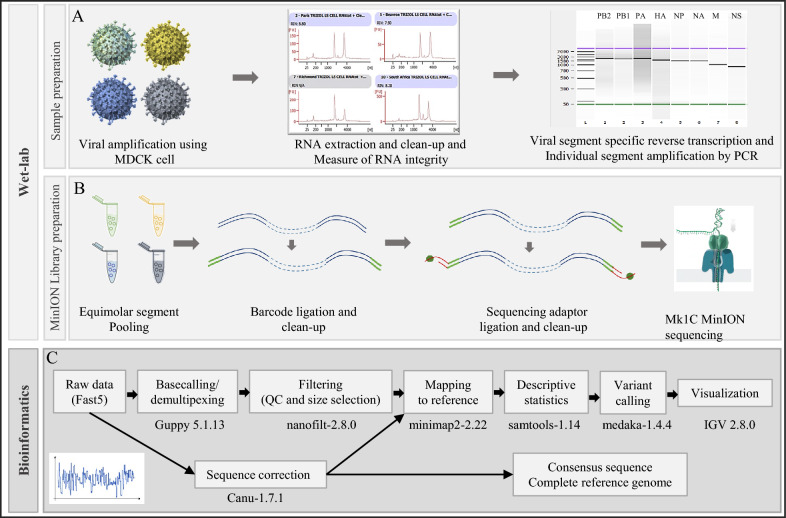
**Schematic workflow implemented for long-read sequencing of equine influenza virus.** The four equine influenza viruses A/equine/Beuvron-en-Auge/2/2009, A/equine/Paris/1/2018, and OIE recommended vaccine strains A/equine/South Africa/4/2003 and A/equine/Richmond/1/2007 were analyzed. **A** After viral amplification in the MDCK cell line and RNA extraction, the eight genomic segments were individually amplified by RT‒PCR. Amplified DNA products were controlled by capillary electrophoresis. **B** For each strain, the eight amplicons were pooled with equimolar ratios, and sequencing libraries were prepared and loaded on a flow cell. **C** The bioinformatics workflow used from raw data to consensus sequence construction. The reference strain is A/equine/Ohio/2005 (GenBank accession numbers: CY067323, CY067324, CY067325, CY067326, CY067327, CY067328, CY067329, CY067330).

The four selected EIV strains A/equine/Beuvron-en-Auge/2/2009 and A/equine/Paris/1/2018 as well as the OIE-recommended vaccine strains A/equine/Richmond/1/2007 and A/equine/South Africa/4/2003 were used to obtain complete amplicon sequences using the long-read sequencing technology developed by Oxford Nanopore Technology. The workflow used is described in Figure 1. Direct RNA sequencing was carried out using the A/equine/South Africa/4/2003 strain to evaluate the relative sensitivity and accuracy of this approach (data not shown).

After size and quality filtering, a mean of 235 222 reads per strain with 158 077 reads for A/equine/South Africa/4/2003, 173 495 reads for A/equine/Richmond/1/2007, 189 296 for A/equine/Paris/1/2018 and 420 018 reads for A/equine/Beuvron-en-Auge/2/2009 were produced (detailed sequencing statistics in Table 1). The average read length was 1291 bp for A/equine/South Africa/4/2003, 1132 bp for A/equine/Richmond/1/2007, 1215 bp for A/equine/Paris/1/2018 and 974 bp for A/equine/Beuvron-en-Auge/2/2009. The average quality (Phred score) for the four strains was Q = 22. For the four strains, a mapping rate varying between 99.81 and 99.97% with full coverage of the eight influenza genome segments was obtained using the reference genome A/equine/Ohio/113461-1/2005.

**Table 1:** **Sequencing statistics**

	South Africa	Richmond	Beuvron	Paris
Total reads	515 999	519 851	976 418	582 633
Filtered reads (Q > 10; Size > 600 bp)				
Reads number	158 077	173 495	420 018	189 296
Average length (nt)	1291	1132	974	1215
Average quality	22.5	22.6	22.1	22.6
Mapped reads				
Total reads mapped	157 979	173 393	419 899	188 939
% reads mapped	99.94%	99.94%	99.97%	99.81%
Segment1 (PB2)	20 197	27 727	80 078	26 715
Segment2 (PB1, PB1-F2)	18 731	26 059	135 601	26 609
Segment3 (PA)	19 554	24 642	31 455	33 794
Segment4 (HA)	17 898	21 245	35 347	28 913
Segment5 (NP)	27 086	16 715	19 096	20 082
Segment6 (NA)	23 350	21 141	29 387	21 860
Segment7 (M1–M2)	26 425	35 844	54 494	35 756
Segment8 (NS1, NEP)	23 634	17 339	47 279	25 569

Detailed sequencing statistics obtained after demultiplexing and size and quality filtering of the sequenced strains A/equine/South Africa/4/2003 (South Africa), A/equine/Richmond/1/2007 (Richmond), A/equine/Beuvron-en-Auge/2/2009 (Beuvron), and A/equine/Paris/1/2018 (Paris).

**Figure 2:**
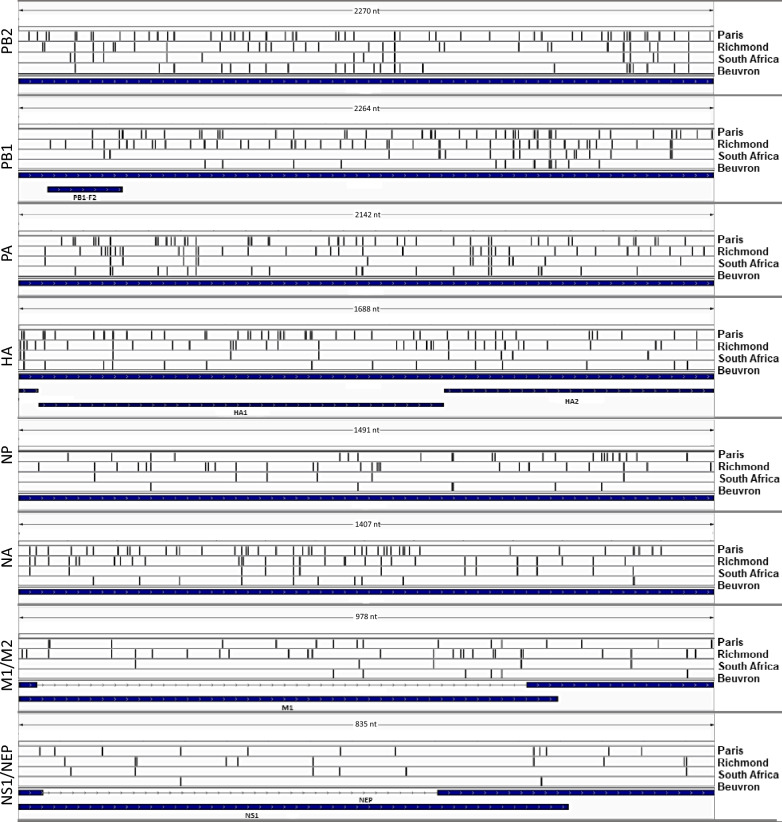
**Nucleotidic variation patterns.** This graphic extracted from Integrative Genomics Viewer [77] depicts variants as vertical bars along the x-axis for the different sequences shown on the y-axis. The four consensus genomic sequences of A/equine/Paris/1/2018 (Paris), A/equine/Richmond/1/2007 (Richmond), A/equine/South Africa/4/2003 (South Africa) and A/equine/Beuvron-en-Auge/2/2009 (Beuvron) are aligned to the reference (A/equine/Ohio/113461-1/2005 sequences) to visualize the variation patterns across the strains. The scale is indicated for each segment.

The nucleotide sequences of the viral genomes of the four strains were compared to those of A/equine/Ohio/113461-1/2005 (Figure 2, Additional files 3 and 4). No nucleotide discrepancies were observed between the genome sequence generated by amplicons and direct RNA sequencing of A/equine/South Africa/4/2003 (data not shown). A total of 538 substitutions for the four strains were detected. The A/equine/Paris/1/2018 genome exhibited a higher number of nucleotide substitutions (287 substitutions), particularly in the HA and NA segments, with 45 substitutions for each. Additionally, higher nucleotide sequence diversity was found in segments 1 and 3, encoding RNA-polymerase (FluPol) subunits PB2 and PA, respectively, with 53 and 49 substitutions among them and 32 and 31 being specific to A/equine/Paris/1/2018.

### Phylogenetic analysis

**Figure 3:**
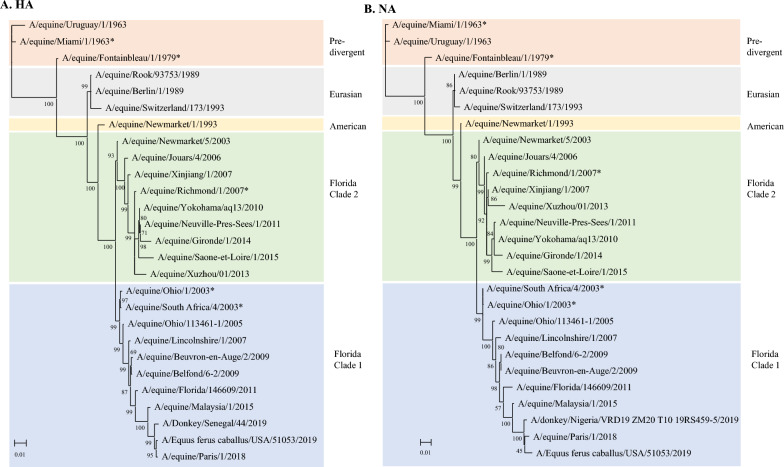
**Phylogenetic analysis of the HA (A) and NA (B) nucleotide sequences for 27 EIV strains.** The analysis includes representative strains of the main lineages, sublineages, and vaccine strains (*). Divergence of lineages are represented by colored background: orange (pre-divergence), grey (Eurasian), yellow (American), green (American sublineage Florida Clade 2) and blue American sublineage Florida Clade 1). Phylogenetic trees were created using the maximum likelihood method and Hasegawa-Kishino-Yano model with 1000 bootstraps.

**Figure 4:**
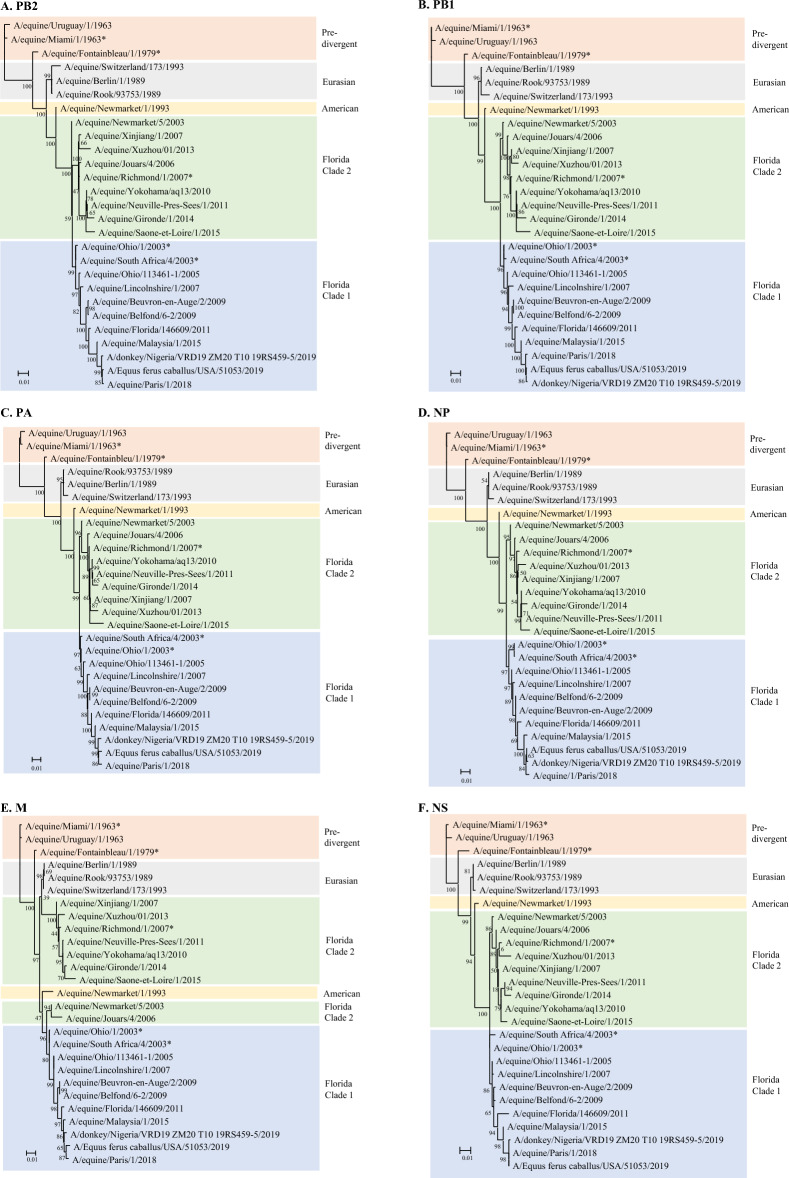
**Phylogenetic analysis of the nucleotide sequences encoding PB2 (A), PB1 (B), PA (C), NP (D), M (E) and NS (F).** The analysis includes representative strains of the main lineages, sublineages, and vaccine strains (*). Divergence of lineages are represented by colored background: orange (pre-divergence), grey (Eurasian), yellow (American), green (American sublineage Florida Clade 2) and blue American sublineage Florida Clade 1). Phylogenetic trees were created using the maximum likelihood method and Hasegawa-Kishino-Yano model with 1000 bootstraps.

Individual phylogenetic trees were constructed for each of the eight segments, including a limited set of mostly European sequences from the literature. The accession numbers of the selected sequences are presented in Additional file 2. Figure 3 shows the analysis of complete HA and NA coding sequences. From 2011, the French isolates were present in both the FC1 and FC2 strains, with the A/equine/Paris/1/2018 HA segment exhibiting a higher phylogenetic distance from the vaccine strains. These observations for the HA gene were correlated with the complete NA sequence analysis. Figure 4 shows the phylogenetic trees of the four segments encoding the components of the influenza ribonucleoprotein complex (with NP and FluPol subunits PA, PB1, and PB2) as well as segments encoding M proteins (M1 and M2) and NS proteins (NS1 and NEP). All the phylogenetic trees correlate well with those of the HA and NA segments.

### Analysis of HA amino acid alignment between circulating and ancestral viruses with vaccine strains

#### The antigenic sites

**Figure 5:**
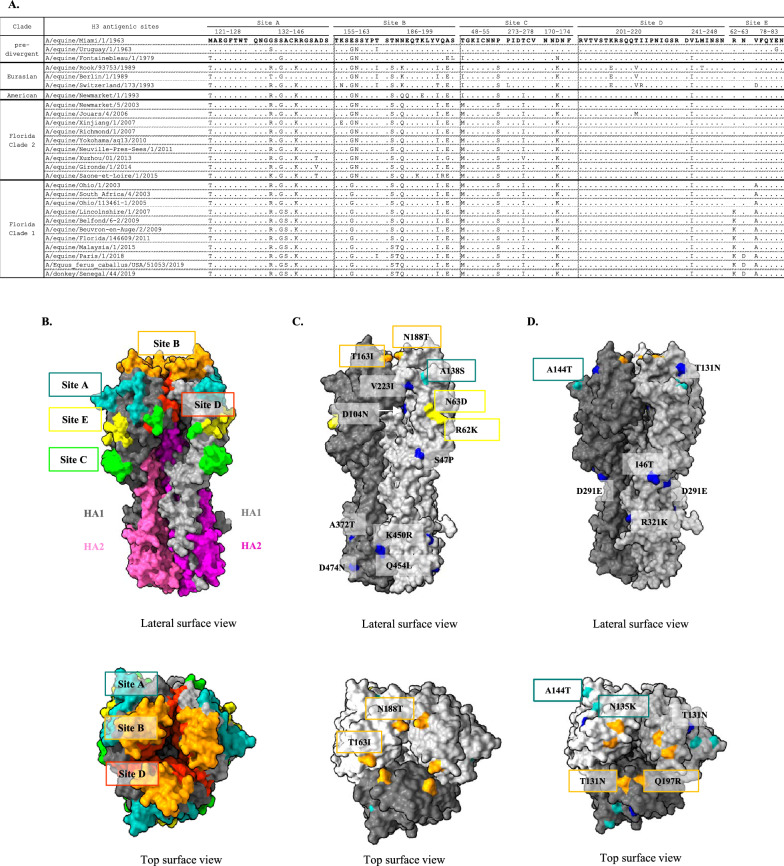
**HA antigenic sites.** **A** Amino acid alignments of the five antigenic sites A to E with HA sequences determined for French strains and other fully sequenced viral strains and compared with A/equine/Miami/1/1963. The antigenic sites defined for the human H3 influenza virus were used as a reference [36, 37, 39]. **B** Lateral and top views of the 3D structure of H3 hemagglutinin (PDB accession number: 4UO0) and location of its antigenic sites. While the HA2 domain (in pink and magenta) constitutes the stem, HA1 domains form the head of the HA bearing the antigenic sites. Antigenic sites are colored in cyan (site A), orange (site B), green (site C), red (site D), and yellow (site E). **C** Location of HA amino acid substitutions between the FC1 strains A/equine/Ohio/1/2003 and A/equine/Paris/1/2018. Amino acid changes are colored according to their positions in the corresponding antigenic sites (as in **B**) or in blue. **D** Location of HA amino acid substitutions between the FC2 strains A/equine/Richmond/1/2007 and A/equine/Saone-et-Loire/1/2015. Color patterning as in **C**.

Five antigenic sites (A–E) have been previously defined on the hemagglutinin of influenza viruses of the H3N2 type in human and have been used for analyses of equine H3N8 (Figure 5 and Additional file 5), [36–39]). Figure 5A shows a multiple alignment of amino acid sequences defining these antigenic sites on a selection of equine H3N8 viruses. Figure 5B highlights the positions of the antigenic sites on the HA 3D structure. The recently circulating virus strains A/equine/Paris/1/2018 and A/equine/Beuvron-en-Auge/2/2009 were included in the analysis, with viruses belonging to FC1 and FC2 with representatives of French EIV strains and vaccine strains currently used in France (A/equine/Ohio/1/2003 and A/equine/Richmond/1/2007). Sequence variation in the antigenic sites was observed for the FC1 and FC2 viruses over 40 years when compared to the two viruses isolated in 1963, A/equine/Miami/1/1963 and A/equine/Uruguay/1/1963. Among the 101 residues constituting the antigenic sites, only 19 and 18 substitutions were identified in A/equine/Paris/1/2018 and A/equine/Saone-et-Loire/1/2015, respectively. When compared with the currently used vaccine strains, only four substitutions (R62K, N63D, A138S and N188T) between FC1 circulating strains and A/equine/Ohio/1/2003 and three (A144T, T192K and Q197R) between FC2 strains A/equine/Saone-et-Loire/1/2015 and A/equine/Richmond/1/2007 were identified.

When restricting the analysis to three predivergent strains (with two of the 1963 years, the date of recognized emergence of H3N8 EIV), twelve amino acid substitutions occurred in the HA antigenic sites, several of them being conserved in subsequent clusters (T48I, M121T, G137G, E158G, S159N, T163I, A198E, and V242I). Others (E82G, G135S, D172N, and S199L) were not conserved among representatives of circulating strains of FC1 and FC2 when they diverged from 2003. Eurasian and American lineages (that emerged in the 1980s) displayed additional common substitutions (P55S, G135R/T, R140K, D172K, T187S, N189Q, and V196I) that were conserved in FC1 and FC2 circulating strains. Others (T48I, K156N, N189K, K207E, and T212V) were only represented in these two lineages. Among them, A/equine/Switzerland/173/1993 (Eurasian lineage) displayed additional specific substitutions (V78D, K156N, I213R, and P273L). A/equine/Newmarket/1/1993 (American lineage) also displayed a specific substitution (K193E). Concerning the FC1 and FC2 strains, T48M appeared to be the unique substitution marking these two sublineages. Other conserved substitutions (compared to the 1963 strains) were previously identified in the American lineage. The S159 variant was found only in the A/equine/Miami/1/1963 strain, and the V78A substitution is a hallmark of the FC1 strains when compared to other strains. As exemplified in Figure 5C, several specific substitutions represented in different FC1 strains are R62K, N63D, A138S and N188T. For FC2 viruses, only one substitution in an antigenic site (A144T) was observed between the vaccine strain (A/equine/Richmond/1/2007) and the A/equine/Saone-et-Loire/1/2015 virus (Figure 5D, [11]).

#### The receptor binding site

Because of the importance of receptor binding by HA in virus transmission and cross-species barriers, the analysis was extended to residues associated with binding to a2,3-linked receptors (Additional file 5). These residues are present on two loops on HA1, the 130-loop, the 220-loop, and the 190-helix [40, 41]. As expected, HA1 G225 and Q226 (220-loop), which are involved in receptor binding, are strictly conserved among all the strains analyzed. E190 and K193 are highly conserved (with two exceptions, E190Q and K193E in A/equine/Newmarket/1/1993). R135 and G137 (in the 130-loop and antigenic site A) exhibited full conservation in FC1 and FC2. Amino acid substitutions in the two loops were also identified in FC1 viruses (A138S and V223I).

#### The membrane fusion machinery

Two amino acid stretches in HA1 (a loop from residue 25 to 35) and HA2 (a-helix A between residues 367 and 384) constitute the fusion subdomain of HA that governs the fusion between cell and viral membranes. A single amino acid substitution, T30S, which was proposed to influence membrane fusion activity through local perturbation of the interactions between these two stretches [40], was identified in all FC1 and FC2 viruses. At position 379, a G379E substitution in several FC1 and FC2 viruses was observed. 3D structures of the HA of a Eurasian virus and an FC2 virus show that the glycine marks a break of the a-helix A [40], thus possibly modulating their fusion properties. The two HAs of the French strains A/equine/Paris/1/2018 and A/equine/Beuvron-en-Auge/2/2009 have a Gly at position 379.

Additional substitutions that are not involved in antigenic sites, receptor binding, or the fusion machinery are reported in Additional file 6.

### Analysis of NA amino acid alignment

**Figure 6:**
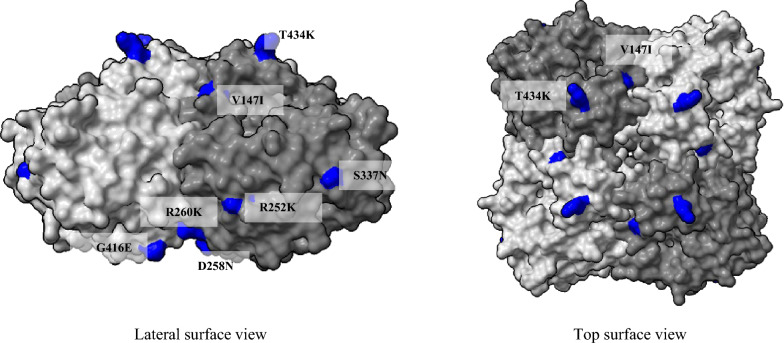
**Positions of the amino acid substitutions on the surface of N8 between the FC1 vaccine strain A/equine/Ohio/1/2003 and the A/equine/Paris/1/2018 strain.** Only the head of NA is represented. Amino acid changes are colored blue. The 3D structure template is the PDB accession number 2HT5.

Fourteen substitutions were identified between A/equine/Paris/1/2018 and the vaccine strain A/equine/Ohio/1/2003, seven in the stalk (A13T, N21S, V35A, G47E, T68I, I74M, R76K) and seven in the head (V147I, R252K, D258N, R260K, S337N, G416E and T434K) (Additional file 6 and 7). Figure 6 shows the substitutions exposed on the surface of the head of NA, one of them (V147I) located near the 150 loop of the active site [42].

### Comparison of the viral proteins of the replicative complex

The amino acid sequences of the FluPol subunits (PA, PB1 and PB2) and NP of the two FC1 strains A/equine/Paris/1/2018 and A/equine/Beuvron-en-Auge/2/2009 were compared with A/equine/Ohio/1/2003 and A/equine/Richmond/1/2007, the two OIE-recommended vaccine strains representing FC1 and FC2, respectively (Table 2). A greater number of changes in the EIV strain A/equine/Paris/1/2018 were identified in comparison to A/equine/Ohio/1/2003. This strain from 2018 possesses eight amino acid substitutions in PA, one in PB1, nine in PB2, and one in NP with A/equine/Ohio/1/2003. Some substitutions were also identified in A/equine/Beuvron-en-Auge/2/2009, such as in PB2 I63V, I398V, V667 and V686I, in PB1 F94L, R584Q and K621R and in PA E237K and T354I. Twenty-two substitutions between these two strains on the FluPol subunits and NP were also identified, exemplifying the continuous accumulation of substitutions between 2009 and 2018 in FC1 strains. Twenty-one substitutions between the two vaccine strains (isolated in 2003 and 2007) and three between A/equine/Ohio/1/2003 and A/equine/South Africa/4/2003 were also observed.

**Table 2:** **Amino acid sequence comparison between the French strains and the OIE-recommended vaccine strains**

Amino acid position	PB2																	PB1													
	63	65	105	251	295	377	395	398	411	660	661	667	684	686	699	731	754	94	114	119	200	203	329	377	578	584	618	621	644	715	754
A/equine/Ohio/1/2003	I	E	T	R	V	A	A	I	I	K	A	V	A	V	K	V	I	F	I	V	V	R	Q	D	K	R	E	K	V	V	R
A/equine/South Africa/4/2003	–	–	–	–	–	T	–	–	–	–	–	–	–	–	–	–	–	–	–	–	–	–	–	–	–	–	–	–	–	–	–
A/equine/Ohio/113461-1/2005	–	–	–	–	–	–	–	V	V	R	–	–	–	–	–	I	–	L	–	–	–	–	–	–	I	–	–	R	–	–	–
A/equine/Richmond/2007	–	–	A	K	–	–	–	V	–	–	–	–	–	–	–	–	–	L	–	M	–	–	R	E	–	–	D	–	–	–	–
A/equine/Beuvron-en-Auge/2/2009	V	–	–	–	I	–	V	V	–	–	–	I	–	I	–	–	–	L	–	–	–	K	–	–	–	Q	–	R	–	–	–
A/equine/Paris/1/2018	V	K	–	–	–	–	–	V	–	–	T	I	T	I	R	–	V	L	V	–	I	–	–	–	–	Q	–	R	I	A	G

	PA																			NP											
Amino acid position	59	64	86	98	210	237	259	321	335	348	354	367	409	465	476	505	538	626	636	136	214	257	359	430	450						
A/equine/Ohio/1/2003	E	E	M	T	T	E	P	S	L	L	T	K	S	I	A	V	E	K	V	M	R	I	T	T	N						
A/equine/South Africa/4/2003	–	–	–	–	–	–	–	–	–	–	–	–	–	–	–	I	–	–	–	–	K	–	–	–	–						
A/equine/Ohio/113461–1/2005	–	–	–	–	–	–	–	–	–	–	–	–	–	V	–	–	–	–	–	–	–	–	–	–	–						
A/equine/Richmond/2007	–	D	I	–	M	K	–	N	–	–	I	–	–	–	T	I	–	R	–	I	–	T	A	–	S						
A/equine/Beuvron-en-Auge/2/2009	–	–	–	A	–	–	S	–	–	I	I	–	N	V	–	–	K	–	I	–	–	–	–	–	–						
A/equine/Paris/1/2018	K	–	–	–	–	K	S	–	I	I	I	R	N	–	–	–	–	–	–	–	–	–	–	I	–						

	M1						M2					NS1										NEP									
Amino acid position	15	80	95	208	214	248	19	59	85	87	89	22	48	66	84	129	156	207	209	210	212	52									
A/equine/Ohio/1/2003	V	V	R	R	Q	M	C	L	D	E	G	F	S	E	V	I	V	H	N	G	P	M									
A/equine/South Africa/4/2003	–	–	–	–	–	–	–	–	–	D	–	V	–	–	–	T	I	–	–	–	–	–									
A/equine/Ohio/113461-1/2005	–	–	–	–	–	–	–	M	–	D	–	–	–	–	–	–	–	–	–	–	–	–									
A/equine/Richmond/2007	I	I	K	K	E	–	–	–	S	D	S	–	I	–	I	–	–	Y	–	–	–	–									
A/equine/Beuvron-en-Auge/2/2009	–	–	–	–	–	–	–	M	G	D	–	–	–	K	–	–	–	–	–	W	–	I									
A/equine/Paris/1/2018	I	–	–	–	–	I	Y	M	–	D	–	–	–	K	–	–	–	N	I	–	S	L									

Amino acid identity to A/equine/Ohio/1/2003 is represented as a dot.

### Comparison of M1, M2, NS1 and NEP proteins

Although eleven substitutions were found (mainly accumulating in M1) between A/equine/Richmond/1/2007 and A/equine/Ohio/1/2003, only ten substitutions were identified between A/equine/Paris/1/2018 and A/equine/Ohio/1/2003 (four in NS1) (Table 2).

### PB1-F2

The analysis of the gene product PB1-F2, encoded by a + 1 reading frame shift of segment 2, showed a large number of substitutions. PB1-F2 is an accessory (nonstructural) protein that presents the highest percentage of substitutions, with twenty-two substitutions for the short versions of PB1-F2 made of 81 amino acids (Table 3). Interestingly, a stretch of nine residues was present at the C-ter of PB1-F2 encoded by all the predivergent strains [43], but only in a single FC2 virus (A/equine/Saone-et-Loire/1/2015) and in four of the eleven FC1 strains analyzed, suggesting that PB1-F2 functions in equine cells do not need these last amino acid stretches. While amino acids that have been described to be associated with pathogenicity (T51 and V56; [44]) are conserved among the analyzed strains, residues involved in the inflammatory response (R75 and R79; [45]) are not systematically present. The S66N substitution was identified in all the PB1-F2s analyzed, except those of the predivergent strains, possibly marking a decrease in virulence [46, 47].

**Table 3:**
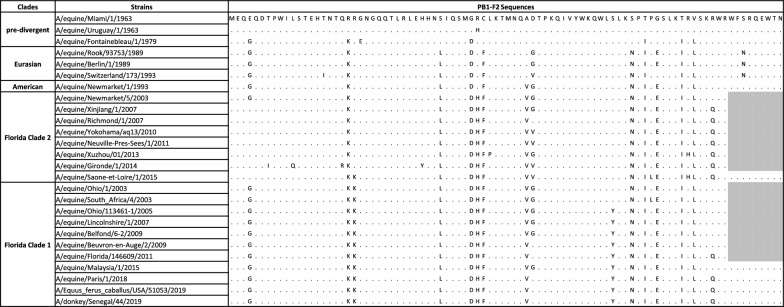
**PB1-F2 amino acid sequence comparison**

The analysis includes the A/equine/Beuvron-en-Auge/2/2009 and A/equine/Paris/1/2018 strains as well as representative strains. Amino acid identity to A/equine/Miami/1/1963 is represented as a dot.

### Functional characterization of equine PB1-F2

**Figure 7:**
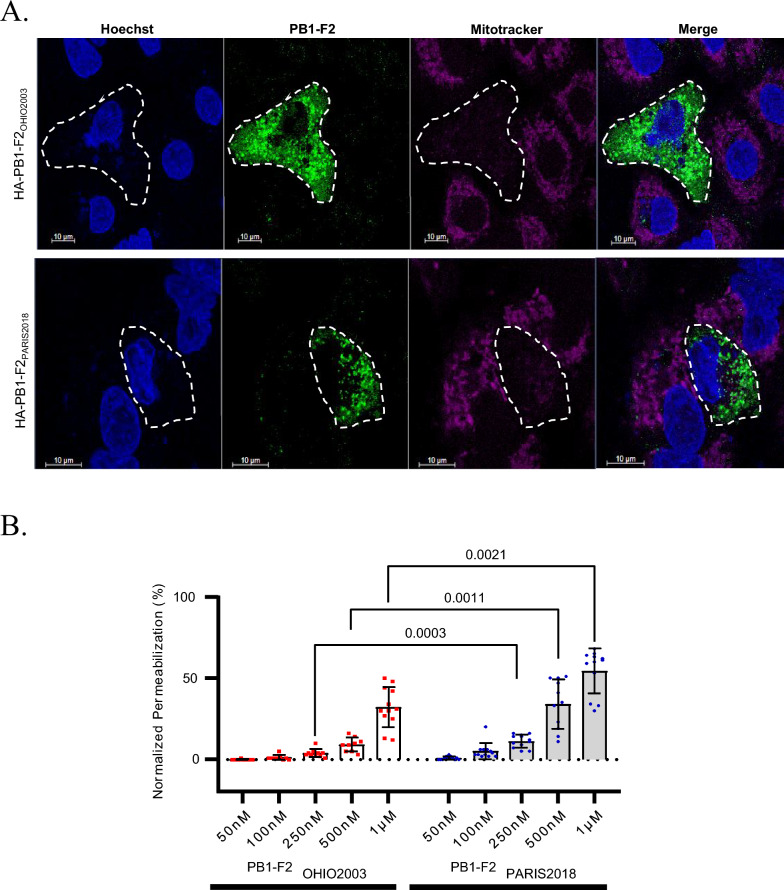
**Comparison of biological properties of the virulence factor PB1-F2 of A/equine/Ohio/1/2003 and A/equine/Paris/1/2018.** **A** Disruption of mitochondrial membrane potential (ΔΨm) in A549 cells expressing HA-tagged PB1-F2 variants from A/equine/Paris/1/2018 (HA-PB1-F2_PARIS2018_) and A/equine/Ohio/1/2003 (HA-PB1-F2_OHIO2003_) viruses. Cells were fixed 48 h post transfection and processed for indirect immunofluorescence staining with an anti-HA-tag rat antibody and an anti-rat secondary antibody coupled with Alexa Fluor 488 (green). Mitochondria were revealed using the ΔΨm-sensitive mitochondrial dye MitoTracker CMX Ros (magenta), and nuclei were revealed with Hoechst (blue). Scale bars, 10 μm. **B** Membrane permeabilization assay using recombinant forms of PB1-F2 encoded by A/equine/Paris/1/2018 (PB1-F2_PARIS2018_) (blue dots) and A/equine/Ohio/1/2003 (PB1-F2_OHIO2003_) (red dots) viruses. LUVs mimicking mitochondrial outer-membrane composition containing the fluorophore probe (ANTS) and quencher (DPX) were incubated with serial dilutions of PB1-F2 forms. The experiment was carried out 4 times in triplicate. Statistical analysis was carried out with REML F(1,99) = 55.01, *P* < 0.0001, and Šídák’s multiple comparison. *P* values are indicated in the figure.

Although PB1-F2 is dispensable for virus replication, it plays significant roles in pathogenesis by altering inflammatory responses, interfering with the host’s innate immune response, and promoting secondary bacterial infections [28, 47–59]. In infected cells, variants of PB1-F2 target mitochondria [50, 53, 60]. Recombinant PB1-F2 has been shown to destabilize and permeabilize synthetic membranes [61–63]. To compare the respective properties of long (90-amino acids long) versus short (81-amino acids long) forms of PB1-F2 of equine viruses, plasmids encoding its A/equine/Paris/1/2018 and the A/equine/Ohio/1/2003 variants were transfected, and their effects on mitochondrial activity were analyzed. Figure 7A shows that the expression of both forms of PB1-F2 resulted in the suppression or lowering of their mitochondrial inner-membrane potential when compared to cells that did not express it, according to a strong decrease in the MitoTracker staining in cells expressing PB1-F2.

To further compare the intrinsic properties of the two variants, a lipid vesicle permeabilization assay was used with large unilamellar vesicles (LUVs) composed of synthetic lipid vesicles mimicking the composition of the outer mitochondrial membrane (OMM) [64]. The two PB1-F2 variants were incubated with LUVs containing a fluorescent soluble probe (ANTS) and its quencher (DPX). The permeabilization of LUVs induced ANTS and DPX efflux, which consequently resulted in dilution and dissociation of the fluorescent probe and its quencher in the extravesicular milieu, as revealed by an increase in ANTS fluorescence. Figure 7B shows that both PB1-F2 variants induced permeabilization of the vesicles in a dose-dependent manner, and the specific permeabilization activity of the A/equine/Paris/1/2018 PB1-F2 variant was twofold higher than that of its homolog (with p-value below 0.0025 for PB1-F2 concentrations 250 nM, 500 nM and 1 μM).

## Discussion

### Whole-genome sequencing

We obtained the complete nucleotide sequence of the A/equine/South Africa/4/2003 virus, both by direct RNA sequencing and by using amplicons. We confirmed that direct RNA sequencing requires a large amount of RNA material, rendering the accuracy of the sequencing difficult to control [29, 65]. Indeed, in our experimental conditions, the accuracy of the direct RNA sequencing was of 68.9% (filtering Q > 7, average Q = 7.8) while indirect sequencing was about 99.95% (filtering Q > 10, average Q = 22.5). Furthermore, indirect sequencing using amplicons by specific influenza genome primers for RT (uni12-RTA) and PCR (see Materials and methods section), allowed the multiplexing of samples by barcoding. This allowed us to pool our four equine influenza strains in a single library preparation, which is currently not possible for direct RNA sequencing. We successfully obtained the whole-genome sequences of four equine influenza viruses using a long-read nanopore sequencer on amplicon RT‒PCR products. This long-read sequencing technology using indirect sequencing is promising as a nomad diagnostic tool, but should be tested using horse nasal swabs and validated for larger number of multiplexed samples.

### Phylogeny

Reassortment events between FC1 and FC2 viruses have been identified and may contribute to evolution [16]. The phylogenetic trees of genomic segments confirmed that A/equine/Paris/1/2018 and A/equine/Beuvron-en-Auge/2/2009 belonged to the EIV H3N8 FC1 (Figures 3 and 4 and [11, 12, 20]) and did not allow the identification of possible segment reassortment events between EIVs.

### Antigenicity

Since 2010, the OIE-ESP has recommended the incorporation of representative EIV strains from both FC1 and FC2 into EI vaccines. Comparison of HA sequences highlights several substitutions between the French EIV strains and the OIE-recommended strain A/equine/Ohio/1/2003 (FC1). The strain A/equine/Paris/1/2018 presents twenty-two substitutions when compared to A/equine/Ohio/1/2003, five of which (A138S, T163I, N188T, R62K, and N63D) are in antigenic sites (site A for the first residue, site B for the two following residues and site E for the last two). The accumulation of these amino acid substitutions within the antibody-binding sites in HA could be sufficient to lead to antigenic drift. As previously observed [20], we also identified one of these substitutions (T163I) only in A/equine/Paris/1/2018 when compared to FC1 and FC2 viruses. According to Wilson and Cox (1990) [66], four or five amino acid substitutions in two separate antigenic sites should be sufficient for escape from preexisting immunity and lead to vaccine failure for human influenza A viruses. For equine influenza A viruses, 10–16 amino acid differences between outbreak and vaccine strains could lead to vaccine breakdown [38, 67]. As previously shown in a large-scale serological study [20], our results suggest that an EIV clade 1 virus, A/equine/Ohio/1/2003, still constitutes an efficient vaccine strain in recent EIV outbreaks, [20]. A similar conclusion could be reached with circulating FC2 EIV and the OIE-recommended vaccine strain A/equine/Richmond/2007, with only 4 substitutions identified in the antigenic sites.

### Equine influenza markers

Equine influenza H3N8 viruses represent a single genetic lineage [68] resulting from the crossover of an avian influenza virus since its first isolation in 1963 [10]. The adaptation of avian influenza A virus to the equine host has been documented, and several host-specific markers have been identified [68, 69]. Comparison between the FluPol, M1, M2, NS1, and NEP sequences of A/equine/Paris/1/2018 and A/equine/Beuvron-en-Auge/2/2009 with representatives of earlier and FC2 strains shows a general conservation of the equine-specific markers with some exceptions. In PB1, a reversion from the recent (since 1997) equine marker I114 was identified in A/equine/Paris/1/2018 (FC1) and A/equine/Saone-et-Loire/1/2015 (FC2) to valine. Additionally, the F94L and K621R substitutions appeared since 2005 in FC1 viruses only. In PA, reversion of the equine E237 to the avian K237 marker has been observed for the most recent Fc1 strain (A/equine/Paris/1/2018) and since 2007 for FC2 strains. This position pertains to a cluster of additional equine-specific markers (positions 213, 216, 217, 231, and 244). S409N substitution was also revealed in A/equine/Paris/1/2018 and A/equine/Beuvron-en-Auge/2/2009, confirming a previously recognized mammal adaptation marker [70] in FC1 viruses [69]. In PB2, the I398V substitution was identified in FC1 viruses in 2005. Similarly, the A684T and A661T substitutions were identified in recent FC1 viruses since 2011 and 2015, respectively. Positions 661 and 684 are known as markers for mammalian adaptation in other influenza viruses [41, 71–74].

### PB1-F2

PB1-F2 is an accessory protein (influenza viruses circulating in humans and other mammalian species do not always encode this polypeptide) that is usually 90 amino acids long whose action is dependent on the viral strain and host species [28, 75]. PB1-F2 displays proinflammatory properties in mammals [45]. In contrast, PB1-F2 seems to attenuate pathogenicity in avian species but extends viral shedding and transmission in chickens [28, 51].

In mice, amino acids L62, R75, R79, and L82 from influenza A viruses were sufficient to generate an inflammatory response. Mutations at these four positions are sufficient to attenuate the pro-inflammatory properties of the protein. It was thus suggested that some PB1-F2 noninflammatory motifs (P62, H75, Q79, and S82) may diminish the risk of secondary bacterial infection [45]. Moreover, it was experimentally validated that the PB1-F2 proinflammatory motif increased morbidity in primary viral infection and enhanced secondary bacterial infection in mice.

Our study as well as [11] shows that the A/equine/Beuvron-en-Auge/2/2009 strain displays a PB1-F2 pro-inflammatory motif (L62, R75, and R79) when compared to the A/equine/Paris/1/2018 virus with only L62 and R75. As the effect of these motifs are extrapolated with studies using human and avian influenza virus strains in mice or chickens, their importance should be studied in the context of H3N8 in equines.

Another marked difference between these two equine influenza PB1-F2 is their length. While that of A/equine/Beuvron-en-Auge/2/2009 is only 81 amino acids long, PB1-F2 encoded by A/equine/Paris/1/2018 is 9 amino acids longer with a sequence pattern alternating charged and hydrophobic residues and a hydrophobic residue at position 82, a tryptophan. Full-length versions of PB1-F2 (predominantly 87 or 90 amino acids) have been reported to specifically translocate into mitochondria through their C-terminal region, which acts as a mitochondrial targeting sequence and induces apoptosis [53, 60, 75, 76]. Our functional analyses (on cellular mitochondria and synthetic membranes) reveal a different behavior of the 81- and 90-amino acid-long PB1-F2. Membrane permeabilization was shown to be more efficient with the longer than with the shorter (81 amino acid long) version of PB1-F2 on synthetic membranes. Both forms were able to block the mitochondrial membrane potential when expressed in the cell cytosol. We thus favor the hypothesis that both the length and the amino acid composition may possibly account for the contribution of PB1-F2 in virulence, a feature that should be validated in an infectious context using recombinant viruses expressing these two forms of PB1-F2 in the same genetic H3N8 backbone.

In conclusion, our study highlights the ongoing evolution of equine influenza viruses, with subtle antigenic changes in hemagglutinin and unique genetic variations notably identified in the A/equine/Paris/1/2018 strain. Furthermore, this strain encodes a full-length accessory protein, PB1-F2, resulting in higher permeabilization capacity when compared to shorter forms and possibly contributing to its virulence. The use of advanced long-read sequencing technologies appears to be imperative for monitoring subtle genetic variabilities of emerging variants to identify key virulence markers in the ever-changing landscape of EIV.

### Supplementary Information

**Additional file 1. Primer sequences for viral genomic segment amplification.**

**Additional file 2. Accession numbers of all selected sequences used for phylogenetic analyses.**

**Additional file 3. Unique and shared nucleotide variations.** This graphic represents each identified variant as a dot along the x-axis, according to the number of strains that contained it along the y-axis. On the right, the bar plot represents the total number of variants that are either unique to a strain (*N* = 1) or shared between two to four of the analyzed strains.

**Additional file 4. Number of nucleotide substitutions.** The reference sequence used was A/equine/Ohio/113461–1/2005. The number of substitutions per segment and by strain is shown in black and strain-specific in gray.

**Additional file 5. Multiple alignment of HA amino acid sequences for selected strains since 1963.** Antigenic sites are indicated in blue outlined boxes. Amino acid identity is represented with a dot. Absent amino acids are represented with a line. Blue letters (A-E) indicate the antigenic sites. The 130-loop, 190-helix, and 220-loop involved in the receptor-binding site are indicated in orange outlined boxes.

**Additional file 6. Substitutions found in HA and NA.** Comparison to A/equine/Ohio/1/2003 for 2009 and 2018 French strains, strain used for MinION consensus sequence, OIE recommended vaccine strains A/equine/South Africa/4/2003 (Fc1) and A/equine/Richmond/1/2007 (Fc2). Numbering according to mature HA. Lines represent identity to A/equine/Ohio/1/2003.

**Additional file 7. Multiple alignment of NA amino acid sequences for selected strains since 1963.** Amino acid identity is represented with a dot. Absent amino acids are represented with a line.

## Data Availability

The datasets used and/or analyzed during the current study are available from the corresponding author upon reasonable request.

## References

[1] Rash A, Morton R, Woodward A, Maes O, Mccauley J, Bryant N, Elton D (2017). Evolution and divergence of H3N8 equine influenza viruses circulating in the United Kingdom from 2013 to 2015. Pathogens.

[2] Cullinane A, Newton JR (2013). Equine influenza-A global perspective. Vet Microbiol.

[3] Chappell DE, Barnett DC, James K, Craig B, Bain F, Gaughan E, Schneider C, Vaala W, Barnum SM, Pusterla N (2023). Voluntary surveillance program for equine influenza virus in the United States during 2008–2021. Pathogens.

[4] Chambers TM (2021) AAET Infectious disease guidelines: Equine influenza virus (EIV)

[5] Dominguez M, Münstermann S, de Guindos I, Timoney P (2016). Equine disease events resulting from international horse movements: systematic review and lessons learned. Equine Vet J.

[6] Cullinane A (2014). Equine influenza and air transport. Equine Vet Educ.

[7] Chambers TM (2022). Equine influenza. Cold Spring Harb Perspect Med.

[8] Singh RK, Dhama K, Karthik K, Khandia R, Munjal A, Khurana SK, Chakraborty S, Malik YS, Virmani N, Singh R, Tripathi BN, Munir M, van der Kolk JH (2018). A comprehensive review on equine influenza virus: etiology, epidemiology, pathobiology, advances in developing diagnostics, vaccines, and control strategies. Front Microbiol.

[9] Webster RG (1993). Are equine 1 influenza viruses still present in horses?. Equine Vet J.

[10] Waddell GH, Teigland MB, Sigel MM (1963). A new influenza virus associated with equine respiratory disease. J Am Vet Med Assoc.

[11] Fougerolle S, Legrand L, Lecouturier F, Sailleau C, Paillot R, Hans A, Pronost S (2017). Genetic evolution of equine influenza virus strains (H3N8) isolated in France from 1967 to 2015 and the implications of several potential pathogenic factors. Virology.

[12] Paillot R, Pitel P-H, Pronost S, Legrand L, Fougerolle S, Jourdan M, Marcillaud-Pitel C (2019). Florida clade 1 equine influenza virus in France. Vet Rec.

[13] Oladunni FS, Oseni SO, Martinez-Sobrido L, Chambers TM (2021). Equine influenza virus and vaccines. Viruses.

[14] Daly JM, Lai ACK, Binns MM, Chambers TM, Barrandeguy M, Mumford JA (1996). Antigenic and genetic evolution of equine H3N8 influenza A viruses. J Gen Virol.

[15] Lai ACK, Chambers TM, Holland RE, Morley PS, Haines DM, Townsend HGG, Barrandeguy M (2001). Diverged evolution of recent equine-2 influenza (H3N8) viruses in the Western Hemisphere. Arch Virol.

[16] Bryant NA, Rash AS, Russell CA, Ross J, Cooke A, Bowman S, MacRae S, Lewis NS, Paillot R, Zanoni R, Meier H, Griffiths LA, Daly JM, Tiwari A, Chambers TM, Newton JR, Elton DM (2009). Antigenic and genetic variations in European and North American equine influenza virus strains (H3N8) isolated from 2006 to 2007. Vet Microbiol.

[17] Bryant NA, Rash AS, Woodward AL, Medcalf E, Helwegen M, Wohlfender F, Cruz F, Herrmann C, Borchers K, Tiwari A, Chambers TM, Newton JR, Mumford JA, Elton DM (2011). Isolation and characterisation of equine influenza viruses (H3N8) from Europe and North America from 2008 to 2009. Vet Microbiol.

[18] Legrand LJ, Pitel P-HY, Marcillaud-Pitel CJ, Cullinane AA, Couroucé AM, Fortier GD, Freymuth FL, Pronost SL (2013). Surveillance of equine influenza viruses through the RESPE network in France from November 2005 to October 2010. Equine Vet J.

[19] Walker-Panse L, Rash A, Huckstep J, Payne S, Blake S, Whitlock F, Elton D, Newton R, Bryant NA (2021). Equine influenza virus surveillance in the United Kingdom from 2019 to 2021. Equine Vet J.

[20] Fougerolle S, Fortier C, Legrand L, Jourdan M, Marcillaud-Pitel C, Pronost S, Paillot R (2019). Success and limitation of equine influenza vaccination: the first incursion in a decade of a Florida clade 1 equine influenza virus that shakes protection despite high vaccine coverage. Vaccines (Basel).

[21] OIE expert surveillance panel on equine influenza vaccine composition, OIE, Paris, 4 April 2019. 10.3390/pathogens5040064 PMC5198164 27897990

[22] Nemoto M, Ohta M, Yamanaka T, Kambayashi Y, Bannai H, Tsujimura K, Yamayoshi S, Kawaoka Y, Cullinane,  (2021). Antigenic differences between equine influenza virus vaccine strains and Florida sublineage clade 1 strains isolated in Europe in 2019. Vet J.

[23] Rash A, Woodward A, Bryant N, McCauley J, Elton D (2014). An efficient genome sequencing method for equine influenza [H3N8] virus reveals a new polymorphism in the PA-X protein. Virol J.

[24] Wang J (2015). MinION nanopore sequencing of an influenza genome. Front Microbiol.

[25] Wüthrich D, Lang D, Müller NF, Neher RA, Stadler T, Egli A (2019). Evaluation of two workflows for whole genome sequencing-based typing of influenza A viruses. J Virol Methods.

[26] Pellegrini F, Buonavoglia A, Omar AH, Diakoudi G, Lucente MS, Odigie AE, Sposato A, Augelli R, Camero M, Decaro N, Elia G, Bányai K, Martella V, Lanave G (2023). A cold case of equine influenza disentangled with nanopore sequencing. Animals.

[27] King J, Harder T, Beer M, Pohlmann A (2020). Rapid multiplex MinION nanopore sequencing workflow for Influenza A viruses. BMC Infect Dis.

[28] Cheung P-HH, Lee T-WT, Chan C-P, Jin D-Y (2020). Influenza A virus PB1-F2 protein: An ambivalent innate immune modulator and virulence factor. J Leukoc Biol.

[29] Keller MW, Rambo-Martin BL, Wilson MM, Ridenour CA, Shepard SS, Stark TJ, Neuhaus EB, Dugan VG, Wentworth DE, Barnes JR (2018). Direct RNA sequencing of the coding complete influenza A virus genome. Sci Rep.

[30] Stecher G, Tamura K, Kumar S (2020). Molecular Evolutionary Genetics Analysis (MEGA) for macOS. Mol Biol Evol.

[31] Tamura K, Stecher G, Kumar S (2021). MEGA11: molecular evolutionary genetics analysis version 11. Mol Biol Evol.

[32] Hasegawa M, Kishino H, Yano T (1985). Dating of the human-ape splitting by a molecular clock of mitochondrial DNA. J Mol Evol.

[33] Felsenstein J (1985). Confidence limits on phylogenies: an approach using the bootstrap. Evolution (N Y).

[34] Madeira F, Pearce M, Tivey ARN, Basutkar P, Lee J, Edbali O, Madhusoodanan N, Kolesnikov A, Lopez R (2022). Search and sequence analysis tools services from EMBL-EBI in 2022. Nucleic Acids Res.

[35] Okonechnikov K, Golosova O, Fursov M (2012). Unipro UGENE: a unified bioinformatics toolkit. Bioinformatics.

[36] Both GW, Sleigh MJ, Cox NJ, Kendal AP (1983). Antigenic drift in influenza virus H3 hemagglutinin from 1968 to 1980: multiple evolutionary pathways and sequential amino acid changes at key antigenic sites. J Virol.

[37] Woodward AL, Rash AS, Blinman D, Bowman S, Chambers TM, Daly JM, Damiani A, Joseph S, Lewis N, McCauley JW, Medcalf L, Mumford J, Newton JR, Tiwari A, Bryant NA, Elton DM (2014). Development of a surveillance scheme for equine influenza in the UK and characterisation of viruses isolated in Europe, Dubai and the USA from 2010–2012. Vet Microbiol.

[38] Woodward A, Rash AS, Medcalf E, Bryant NA, Elton DM (2015). Using epidemics to map H3 equine influenza virus determinants of antigenicity. Virology.

[39] Lee K, Pusterla N, Barnum SM, Lee D, Martínez-López B (2022). Genome-informed characterisation of antigenic drift in the haemagglutinin gene of equine influenza strains circulating in the United States from 2012 to 2017. Transbound Emerg Dis.

[40] Collins PJ, Vachieri SG, Haire LF, Ogrodowicz RW, Martin SR, Walker PA, Xiong X, Gamblin SJ, Skehel JJ (2014). Recent evolution of equine influenza and the origin of canine influenza. Proc Natl Acad Sci U S A.

[41] Wen F, Blackmon S, Olivier AK, Li L, Guan M, Sun H, Wang PG, Wan XF (2018). Mutation W222L at the receptor binding site of hemagglutinin could facilitate viral adaption from equine influenza A(H3N8) virus to dogs. J Virol.

[42] Yang H, Carney PJ, Mishin VP, Guo Z, Chang JC, Wentworth DE, Gubareva LV, Stevens J (2016). Molecular characterizations of surface proteins hemagglutinin and neuraminidase from recent H5Nx avian influenza viruses. J Virol.

[43] Lu G, Guo W, Qi T, Ma J, Zhao S, Tian Z, Pan J, Zhu C, Wang X, Xiang W (2013). Genetic analysis of the PB1-F2 gene of equine influenza virus. Virus Genes.

[44] Marjuki H, Scholtissek C, Franks J, Negovetich NJ, Aldridge JR, Salomon R, Finkelstein D, Webster RG (2010). Three amino acid changes in PB1-F2 of highly pathogenic H5N1 avian influenza virus affect pathogenicity in mallard ducks. Arch Virol.

[45] Alymova IV, Green AM, van de Velde N, McAuley JL, Boyd KL, Ghoneim HE, McCullers JA (2011). Immunopathogenic and antibacterial effects of H3N2 influenza A virus PB1-F2 map to amino acid residues 62, 75, 79, and 82. J Virol.

[46] Conenello GM, Zamarin D, Perrone LA, Tumpey T, Palese P (2007). A single mutation in the PB1-F2 of H5N1 (HK/97) and 1918 influenza A viruses contributes to increased virulence. PLoS Pathog.

[47] Varga ZT, Ramos I, Hai R, Schmolke M, García-Sastre A, Fernandez-Sesma A, Palese P (2011). The influenza virus protein PB1-F2 inhibits the induction of type i interferon at the level of the MAVS adaptor protein. PLoS Pathog.

[48] Alymova IV, Samarasinghe A, Vogel P, Green AM, Weinlich R, McCullers JA (2014). A novel cytotoxic sequence contributes to influenza A viral protein PB1-F2 pathogenicity and predisposition to secondary bacterial infection. J Virol.

[49] Dudek SE, Wixler L, Nordhoff C, Nordmann A, Anhlan D, Wixler V, Ludwig S (2011). The influenza virus PB1-F2 protein has interferon antagonistic activity. Biol Chem.

[50] Wang R, Zhu Y, Ren C, Yang S, Tian S, Chen H, Jin M, Zhou H (2021). Influenza A virus protein PB1-F2 impairs innate immunity by inducing mitophagy. Autophagy.

[51] James J, Howard W, Iqbal M, Nair VK, Barclay WS, Shelton H (2016). Influenza A virus PB1-F2 protein prolongs viral shedding in chickens lengthening the transmission window. J Gen Virol.

[52] Le Goffic R, Bouguyon E, Chevalier C, Vidic J, Da Costa B, Leymarie O, Bourdieu C, Decamps L, Dhorne-Pollet S, Delmas B (2010). Influenza A virus protein PB1-F2 exacerbates IFN-β expression of human respiratory epithelial cells. J Immunol.

[53] Yoshizumi T, Ichinohe T, Sasaki O, Otera H, Kawabata SI, Mihara K, Koshiba T (2014). Influenza a virus protein PB1-F2 translocates into mitochondria via Tom40 channels and impairs innate immunity. Nat Commun.

[54] McAuley JL, Tate MD, MacKenzie-Kludas CJ, Pinar A, Zeng W, Stutz A, Latz E, Brown LE, Mansell A (2013). Activation of the NLRP3 inflammasome by IAV virulence protein PB1-F2 contributes to severe pathophysiology and disease. PLoS Pathog.

[55] Pinar A, Dowling JK, Bitto NJ, Robertson AAB, Latz E, Stewart CR, Drummond GR, Cooper MA, McAuley JL, Tate MD, Mansell A (2017). PB1-F2 peptide derived from avian influenza A virus H7N9 induces inflammation via activation of the NLRP3 inflammasome. J Biol Chem.

[56] McAuley JL, Hornung F, Boyd KL, Smith AM, McKeon R, Bennink J, Yewdell JW, McCullers JA (2007). Expression of the 1918 influenza A virus PB1-F2 enhances the pathogenesis of viral and secondary bacterial pneumonia. Cell Host Microbe.

[57] Mazel-Sanchez B, Boal-Carvalho I, Silva F, Dijkman R, Schmolke M (2018). H5N1 influenza A virus PB1-F2 relieves HAX-1-mediated restriction of avian virus polymerase PA in human lung cells. J Virol.

[58] Gibbs JS, Malide D, Hornung F, Bennink JR, Yewdell JW (2003). The influenza A virus PB1-F2 protein targets the inner mitochondrial membrane via a predicted basic amphipathic helix that disrupts mitochondrial function. J Virol.

[59] Cheung P-HH, Ye Z-W, Lee T-WT, Chen H, Chan C-P, Jin D-Y (2020). PB1-F2 protein of highly pathogenic influenza A (H7N9) virus selectively suppresses RNA-induced NLRP3 inflammasome activation through inhibition of MAVS-NLRP3 interaction. J Leukoc Biol.

[60] Yamada H, Chounan R, Higashi Y, Kurihara N, Kido H (2004). Mitochondrial targeting sequence of the influenza A virus PB1-F2 protein and its function in mitochondria. FEBS Lett.

[61] Chanturiya AN, Basañez G, Schubert U, Henklein P, Yewdell JW, Zimmerberg J (2004). PB1-F2, an influenza A virus-encoded proapoptotic mitochondrial protein, creates variably sized pores in planar lipid membranes. J Virol.

[62] Vidic J, Richard C-A, Péchoux C, Da Costa B, Bertho N, Mazerat S, Delmas B, Chevalier C (2016). Amyloid assemblies of influenza A virus PB1-F2 protein damage membrane and induce cytotoxicity. J Biol Chem.

[63] Chevalier C, Al Bazzal A, Vidic J, Février V, Bourdieu C, Bouguyon E, Le Goffic R, Vautherot JF, Bernard J, Moudjou M, Noinville S, Chich JF, Da Costa B, Rezaei H, Delmas B (2010). PB1-F2 influenza A virus protein adopts a β-sheet conformation and forms amyloid fibers in membrane environments. J Biol Chem.

[64] Konar S, Arif H, Allolio C (2023). Mitochondrial membrane model: lipids, elastic properties, and the changing curvature of cardiolipin. Biophys J.

[65] Smith MA, Ersavas T, Ferguson JM, Liu H, Lucas MC, Begik O, Bojarski L, Barton K, Novoa EM (2020). Molecular barcoding of native RNAs using nanopore sequencing and deep learning. Genome Res.

[66] Wilson IA, Cox NJ (1990). Structural basis of immune recognition of influenza virus hemagglutinin. Annu Rev Immunol.

[67] Ito M, Nagai M, Hayakawa Y, Komae H, Murakami N, Yotsuya S, Asakura S, Sakoda Y, Kida H (2008). Genetic analyses of an H3N8 influenza virus isolate, causative strain of the outbreak of equine influenza at the Kanazawa racecourse in japan in 2007. J Vet Med Sci.

[68] Mucha V, Holly J, Vareckova E, Kostolansky F (2018). Avian influenza A virus adaptation to the equine host and identification of host-specific markers. Acta Virol.

[69] Murcia PR, Wood JLN, Holmes EC (2011). Genome-scale evolution and phylodynamics of equine H3N8 influenza A virus. J Virol.

[70] Finkelstein DB, Mukatira S, Mehta PK, Obenauer JC, Su X, Webster RG, Naeve CW (2007). Persistent host markers in pandemic and H5N1 influenza viruses. J Virol.

[71] Miotto O, Heiny A, Tan TW, August JT, Brusic V (2008). Identification of human-to-human transmissibility factors in PB2 proteins of influenza A by large-scale mutual information analysis. BMC Bioinformatics.

[72] Miotto O, Heiny AT, Albrecht R, García-Sastre A, Tan TW, August JT, Brusic V (2010). Complete-proteome mapping of human influenza A adaptive mutations: implications for human transmissibility of zoonotic strains. PLoS ONE.

[73] Tamuri AU, dos Reis M, Hay AJ, Goldstein RA (2009). Identifying changes in selective constraints: host shifts in influenza. PLoS Comput Biol.

[74] Hayashi T, Wills S, Bussey KA, Takimoto T (2015). Identification of influenza A virus PB2 residues involved in enhanced polymerase activity and virus growth in mammalian cells at low temperatures. J Virol.

[75] Chen W, Calvo PA, Malide D, Gibbs J, Schubert U, Bacik I, Basta S, O'Neill R, Schickli J, Palese P, Henklein P, Bennink JR, Yewdell JW (2001). A novel influenza A virus mitochondrial protein that induces cell death. Nat Med.

[76] Cheng YY, Yang SR, Wang YT, Lin YH, Chen CJ (2017). Amino acid residues 68–71 contribute to influenza A virus PB1-F2 protein stability and functions. Front Microbiol.

[77] Robinson JT, Thorvaldsdóttir H, Winckler W, Guttman M, Lander ES, Getz G, Mesirov JP (2011). Integrative genomics viewer. Nat Biotechnol.

