# Supplementary material for: Genomic characterization of equine influenza A subtype H3N8 viruses by long read sequencing and functional analyses of the PB1-F2 virulence factor of A/equine/Paris/1/2018
Source: Vet Res. 2024 Mar 22;55:36. doi: 10.1186/s13567-024-01289-8 (PMC10960481; doi:10.1186/s13567-024-01289-8)
Supplement: Supplementary file 1 — Additional file 1. Primer sequences for viral genomic segment amplification. [file 13567_2024_1289_MOESM1_ESM.pptx]

## Slide 1
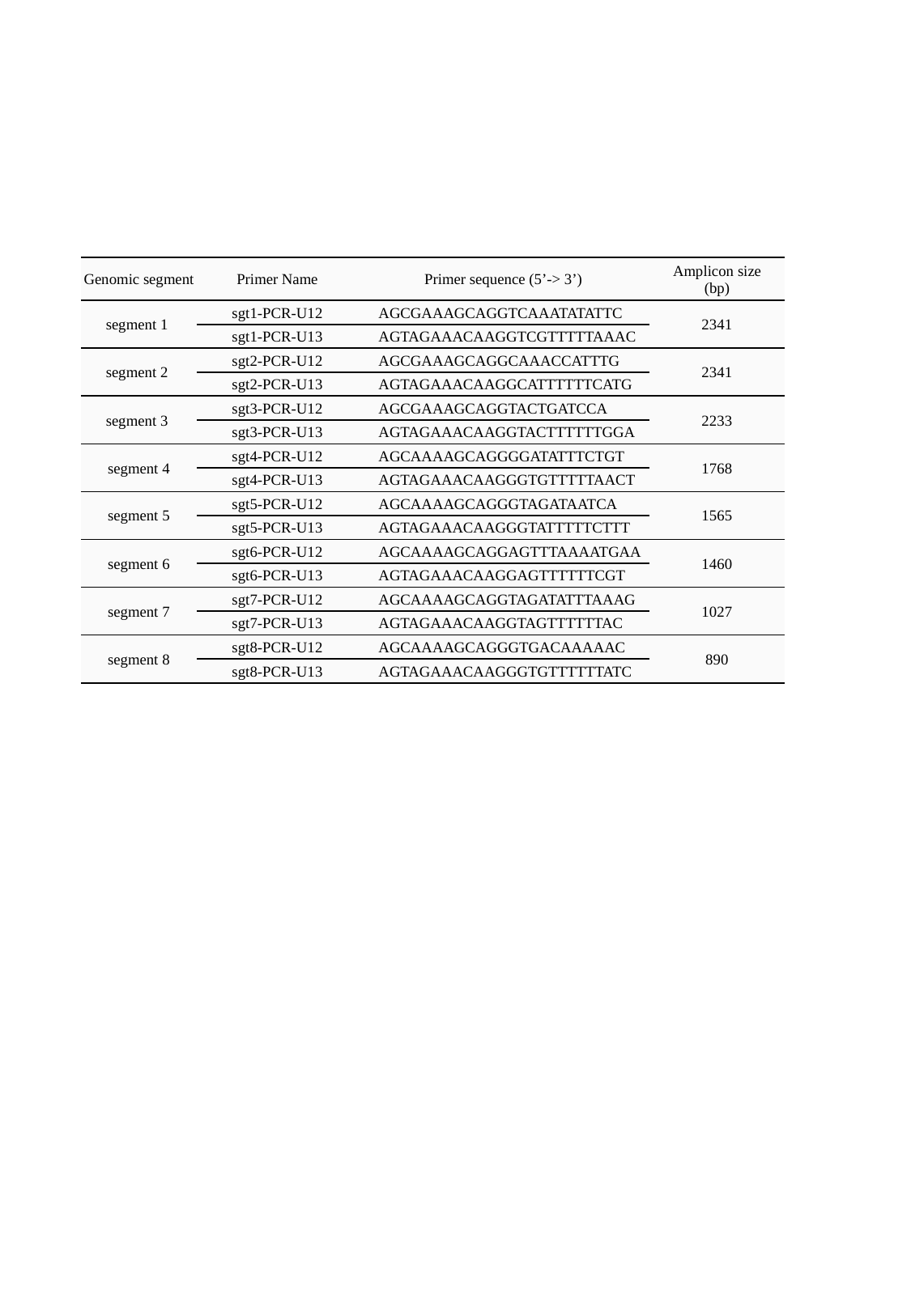

| Genomic segment | Primer Name | Primer sequence (5’-> 3’) | Amplicon size (bp) |
| --- | --- | --- | --- |
| segment 1 | sgt1-PCR-U12 | AGCGAAAGCAGGTCAAATATATTC | 2341 |
| | sgt1-PCR-U13 | AGTAGAAACAAGGTCGTTTTTAAAC | |
| segment 2 | sgt2-PCR-U12 | AGCGAAAGCAGGCAAACCATTTG | 2341 |
| | sgt2-PCR-U13 | AGTAGAAACAAGGCATTTTTTCATG | |
| segment 3 | sgt3-PCR-U12 | AGCGAAAGCAGGTACTGATCCA | 2233 |
| | sgt3-PCR-U13 | AGTAGAAACAAGGTACTTTTTTGGA | |
| segment 4 | sgt4-PCR-U12 | AGCAAAAGCAGGGGATATTTCTGT | 1768 |
| | sgt4-PCR-U13 | AGTAGAAACAAGGGTGTTTTTAACT | |
| segment 5 | sgt5-PCR-U12 | AGCAAAAGCAGGGTAGATAATCA | 1565 |
| | sgt5-PCR-U13 | AGTAGAAACAAGGGTATTTTTCTTT | |
| segment 6 | sgt6-PCR-U12 | AGCAAAAGCAGGAGTTTAAAATGAA | 1460 |
| | sgt6-PCR-U13 | AGTAGAAACAAGGAGTTTTTTCGT | |
| segment 7 | sgt7-PCR-U12 | AGCAAAAGCAGGTAGATATTTAAAG | 1027 |
| | sgt7-PCR-U13 | AGTAGAAACAAGGTAGTTTTTTAC | |
| segment 8 | sgt8-PCR-U12 | AGCAAAAGCAGGGTGACAAAAAC | 890 |
| | sgt8-PCR-U13 | AGTAGAAACAAGGGTGTTTTTTATC | |
