# Supplementary material for: Genomic characterization of equine influenza A subtype H3N8 viruses by long read sequencing and functional analyses of the PB1-F2 virulence factor of A/equine/Paris/1/2018
Source: Vet Res. 2024 Mar 22;55:36. doi: 10.1186/s13567-024-01289-8 (PMC10960481; doi:10.1186/s13567-024-01289-8)
Supplement: Supplementary file 2 — Additional file 2. Accession numbers of all selected sequences used for phylogenetic analyses. [file 13567_2024_1289_MOESM2_ESM.pptx]

## Slide 1
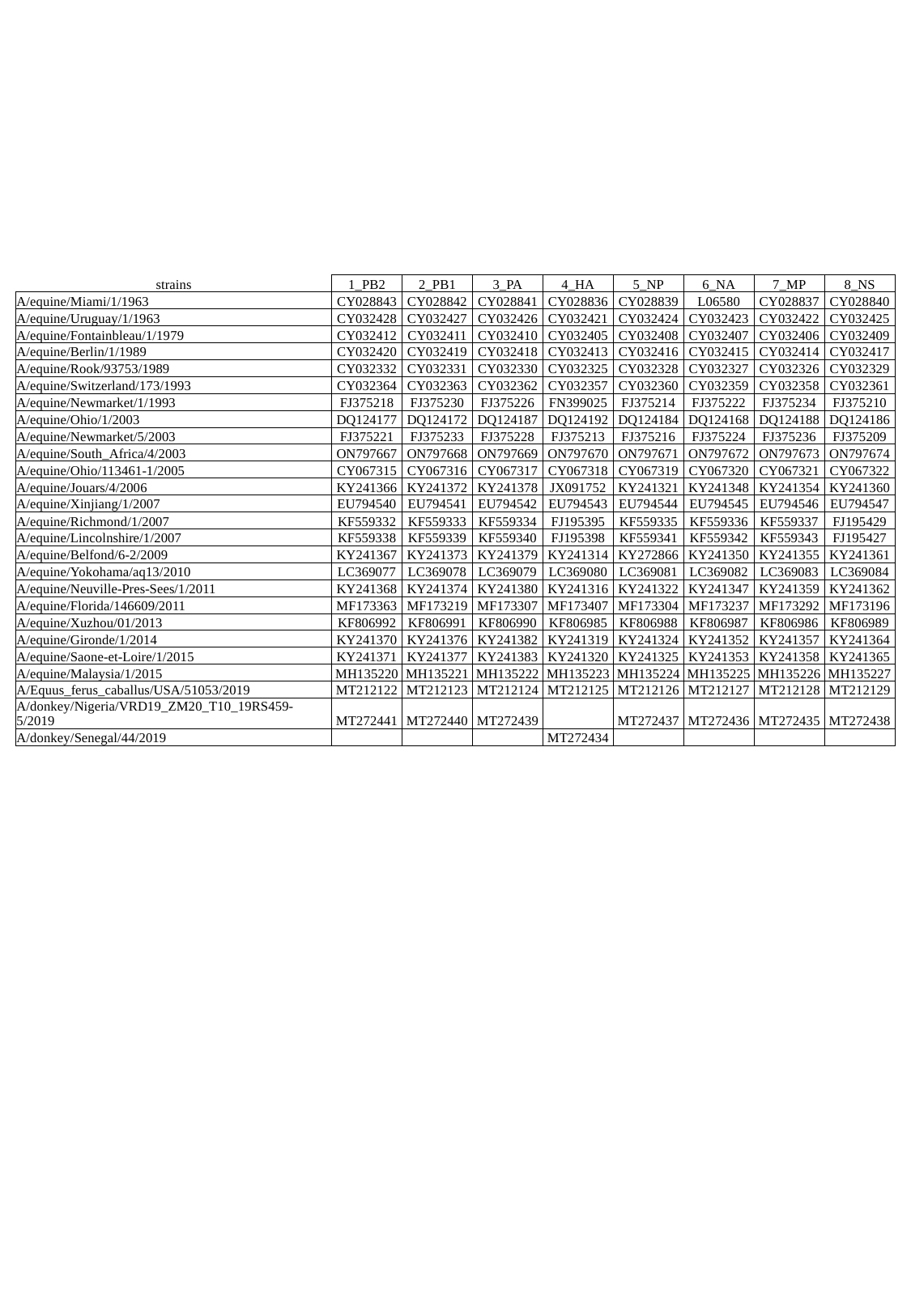

| strains | 1\_PB2 | 2\_PB1 | 3\_PA | 4\_HA | 5\_NP | 6\_NA | 7\_MP | 8\_NS |
| --- | --- | --- | --- | --- | --- | --- | --- | --- |
| A/equine/Miami/1/1963 | CY028843 | CY028842 | CY028841 | CY028836 | CY028839 | L06580 | CY028837 | CY028840 |
| A/equine/Uruguay/1/1963 | CY032428 | CY032427 | CY032426 | CY032421 | CY032424 | CY032423 | CY032422 | CY032425 |
| A/equine/Fontainbleau/1/1979 | CY032412 | CY032411 | CY032410 | CY032405 | CY032408 | CY032407 | CY032406 | CY032409 |
| A/equine/Berlin/1/1989 | CY032420 | CY032419 | CY032418 | CY032413 | CY032416 | CY032415 | CY032414 | CY032417 |
| A/equine/Rook/93753/1989 | CY032332 | CY032331 | CY032330 | CY032325 | CY032328 | CY032327 | CY032326 | CY032329 |
| A/equine/Switzerland/173/1993 | CY032364 | CY032363 | CY032362 | CY032357 | CY032360 | CY032359 | CY032358 | CY032361 |
| A/equine/Newmarket/1/1993 | FJ375218 | FJ375230 | FJ375226 | FN399025 | FJ375214 | FJ375222 | FJ375234 | FJ375210 |
| A/equine/Ohio/1/2003 | DQ124177 | DQ124172 | DQ124187 | DQ124192 | DQ124184 | DQ124168 | DQ124188 | DQ124186 |
| A/equine/Newmarket/5/2003 | FJ375221 | FJ375233 | FJ375228 | FJ375213 | FJ375216 | FJ375224 | FJ375236 | FJ375209 |
| A/equine/South\_Africa/4/2003 | ON797667 | ON797668 | ON797669 | ON797670 | ON797671 | ON797672 | ON797673 | ON797674 |
| A/equine/Ohio/113461-1/2005 | CY067315 | CY067316 | CY067317 | CY067318 | CY067319 | CY067320 | CY067321 | CY067322 |
| A/equine/Jouars/4/2006 | KY241366 | KY241372 | KY241378 | JX091752 | KY241321 | KY241348 | KY241354 | KY241360 |
| A/equine/Xinjiang/1/2007 | EU794540 | EU794541 | EU794542 | EU794543 | EU794544 | EU794545 | EU794546 | EU794547 |
| A/equine/Richmond/1/2007 | KF559332 | KF559333 | KF559334 | FJ195395 | KF559335 | KF559336 | KF559337 | FJ195429 |
| A/equine/Lincolnshire/1/2007 | KF559338 | KF559339 | KF559340 | FJ195398 | KF559341 | KF559342 | KF559343 | FJ195427 |
| A/equine/Belfond/6-2/2009 | KY241367 | KY241373 | KY241379 | KY241314 | KY272866 | KY241350 | KY241355 | KY241361 |
| A/equine/Yokohama/aq13/2010 | LC369077 | LC369078 | LC369079 | LC369080 | LC369081 | LC369082 | LC369083 | LC369084 |
| A/equine/Neuville-Pres-Sees/1/2011 | KY241368 | KY241374 | KY241380 | KY241316 | KY241322 | KY241347 | KY241359 | KY241362 |
| A/equine/Florida/146609/2011 | MF173363 | MF173219 | MF173307 | MF173407 | MF173304 | MF173237 | MF173292 | MF173196 |
| A/equine/Xuzhou/01/2013 | KF806992 | KF806991 | KF806990 | KF806985 | KF806988 | KF806987 | KF806986 | KF806989 |
| A/equine/Gironde/1/2014 | KY241370 | KY241376 | KY241382 | KY241319 | KY241324 | KY241352 | KY241357 | KY241364 |
| A/equine/Saone-et-Loire/1/2015 | KY241371 | KY241377 | KY241383 | KY241320 | KY241325 | KY241353 | KY241358 | KY241365 |
| A/equine/Malaysia/1/2015 | MH135220 | MH135221 | MH135222 | MH135223 | MH135224 | MH135225 | MH135226 | MH135227 |
| A/Equus\_ferus\_caballus/USA/51053/2019 | MT212122 | MT212123 | MT212124 | MT212125 | MT212126 | MT212127 | MT212128 | MT212129 |
| A/donkey/Nigeria/VRD19\_ZM20\_T10\_19RS459-5/2019 | MT272441 | MT272440 | MT272439 | | MT272437 | MT272436 | MT272435 | MT272438 |
| A/donkey/Senegal/44/2019 | | | | MT272434 | | | | |
