# Supplementary material for: Genomic characterization of equine influenza A subtype H3N8 viruses by long read sequencing and functional analyses of the PB1-F2 virulence factor of A/equine/Paris/1/2018
Source: Vet Res. 2024 Mar 22;55:36. doi: 10.1186/s13567-024-01289-8 (PMC10960481; doi:10.1186/s13567-024-01289-8)
Supplement: Supplementary file 3 — Additional file 3. Unique and shared nucleotide variations. This graphic represents each identified variant as a dot along the x-axis, according to the number of strains that contained it along the y-axis. On the right, the bar plot represents the total number of variants that are either unique to a strain (N = 1) or shared between two to four of the analyzed strains. [file 13567_2024_1289_MOESM3_ESM.pptx]

## Slide 1
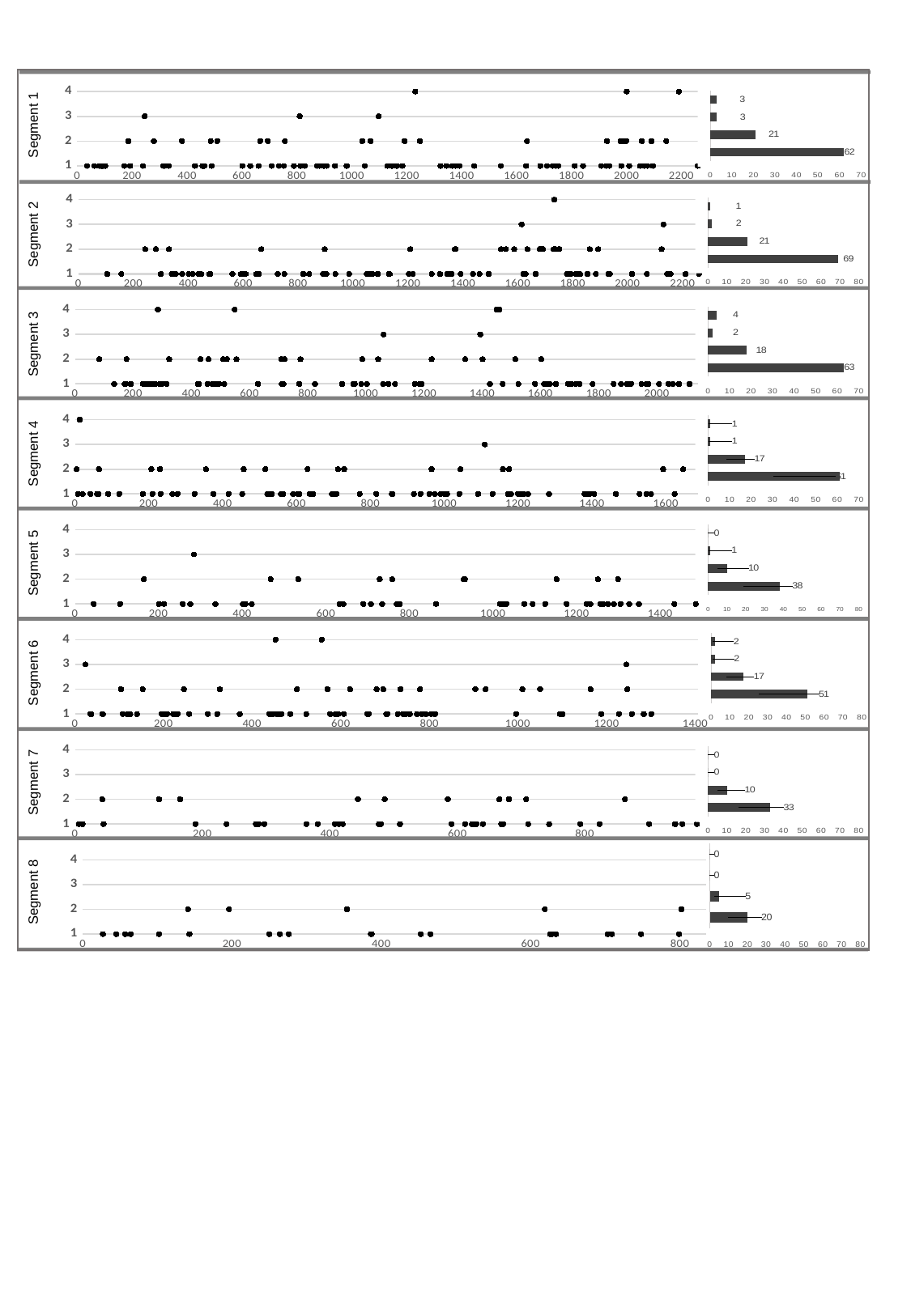

| Segment 1 |
| --- |
| Segment 2 |
| Segment 3 |
| Segment 4 |
| Segment 5 |
| Segment 6 |
| Segment 7 |
| Segment 8 |
### Chart
| Category | |
|---|---|
### Chart
| Category | |
|---|---|
### Chart
| Category | |
|---|---|
### Chart
| Category | |
|---|---|
### Chart
| Category | |
|---|---|
### Chart
| Category | |
|---|---|
### Chart
| Category | |
|---|---|
### Chart
| Category | |
|---|---|
### Chart
| Category | |
|---|---|
### Chart
| Category | |
|---|---|
### Chart
| Category | |
|---|---|
### Chart
| Category | |
|---|---|
### Chart
| Category | |
|---|---|
### Chart
| Category | |
|---|---|
### Chart
| Category | |
|---|---|
### Chart
| Category | |
|---|---|
