# Supplementary material for: Genomic characterization of equine influenza A subtype H3N8 viruses by long read sequencing and functional analyses of the PB1-F2 virulence factor of A/equine/Paris/1/2018
Source: Vet Res. 2024 Mar 22;55:36. doi: 10.1186/s13567-024-01289-8 (PMC10960481; doi:10.1186/s13567-024-01289-8)
Supplement: Supplementary file 4 — Additional file 4. Number of nucleotide substitutions. The reference sequence used was A/equine/Ohio/113461–1/2005. The number of substitutions per segment and by strain is shown in black and strain-specific in gray. [file 13567_2024_1289_MOESM4_ESM.pptx]

## Slide 1
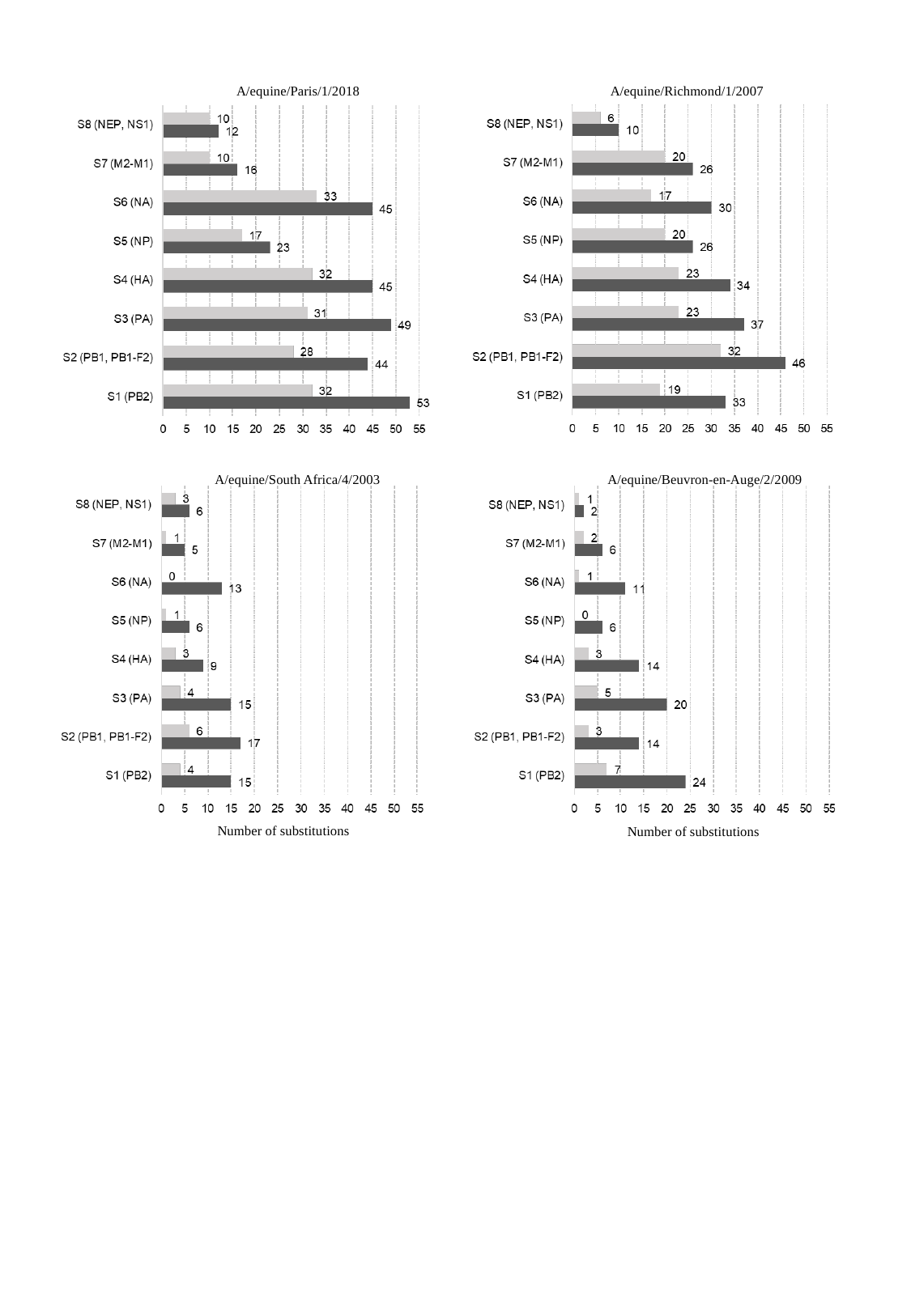

A/equine/Paris/1/2018
A/equine/Richmond/1/2007
A/equine/South Africa/4/2003
A/equine/Beuvron-en-Auge/2/2009
Number of substitutions
Number of substitutions
