# Supplementary figures and images for: Genomic characterization of equine influenza A subtype H3N8 viruses by long read sequencing and functional analyses of the PB1-F2 virulence factor of A/equine/Paris/1/2018
Source: Vet Res. 2024 Mar 22;55:36. doi: 10.1186/s13567-024-01289-8 (PMC10960481; doi:10.1186/s13567-024-01289-8)

## Slide 1
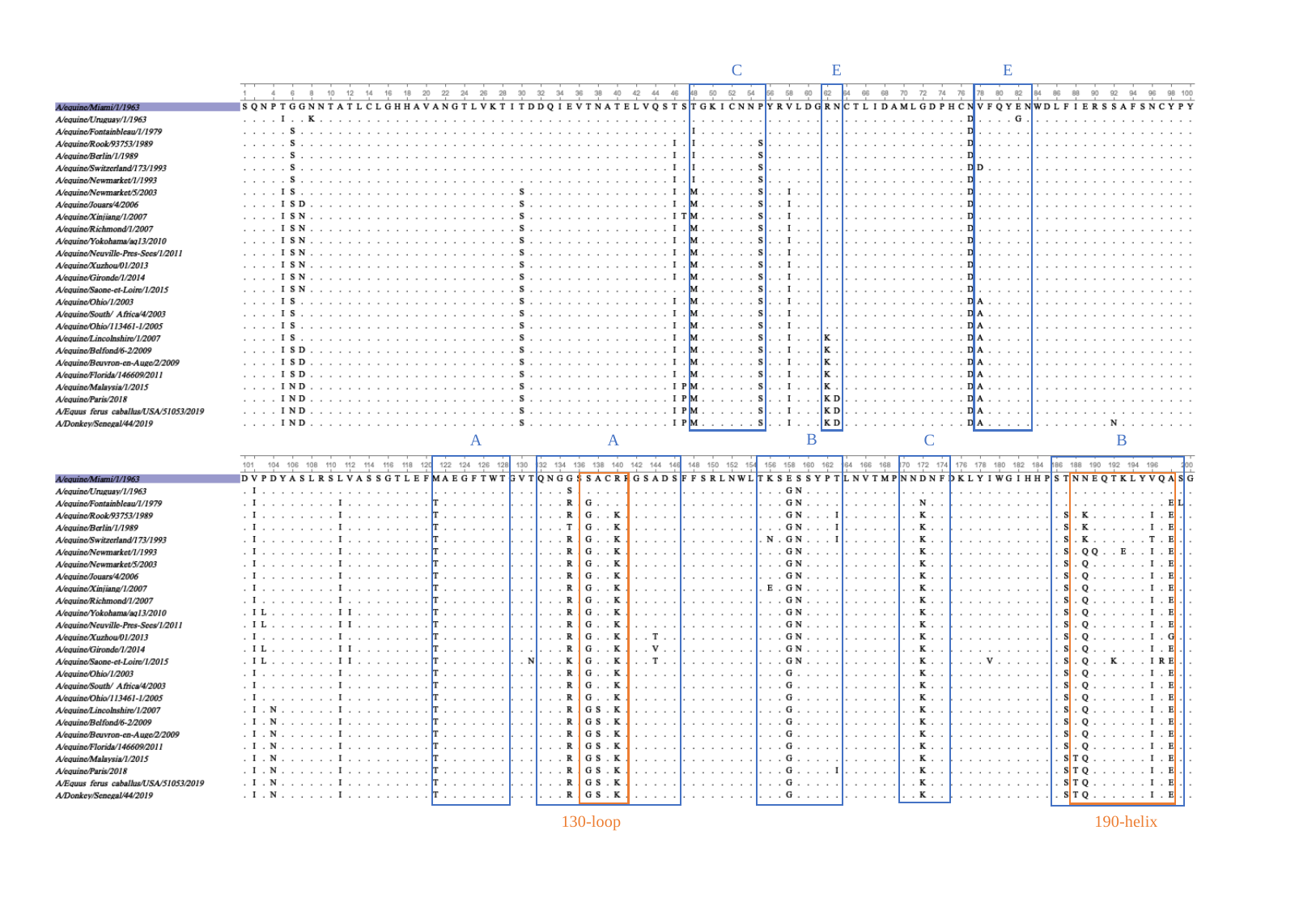

E
E
A
A
B
B
C
C
190-helix
130-loop

## Slide 2
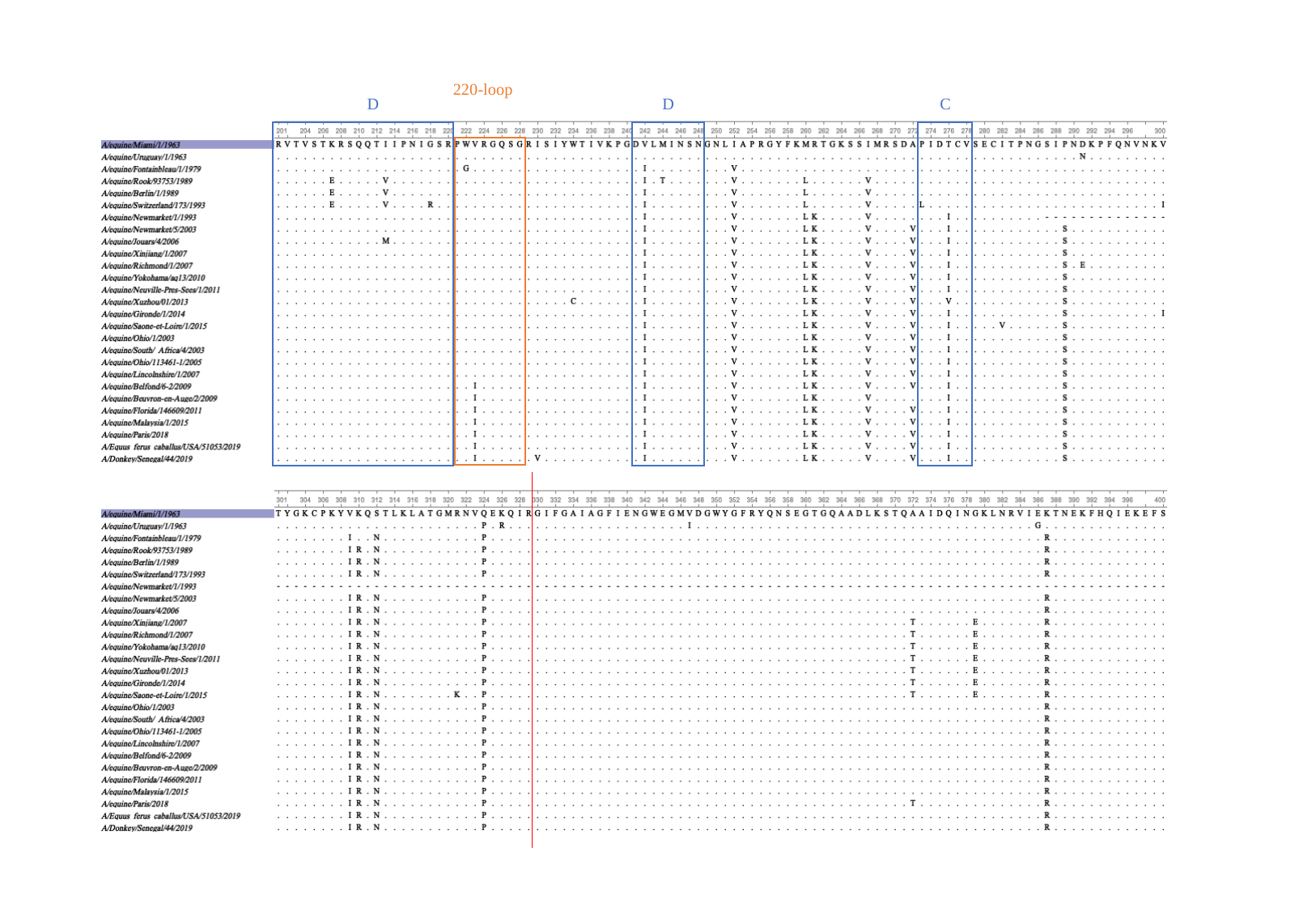

D
D
C
220-loop
HA2
HA1

## Slide 3
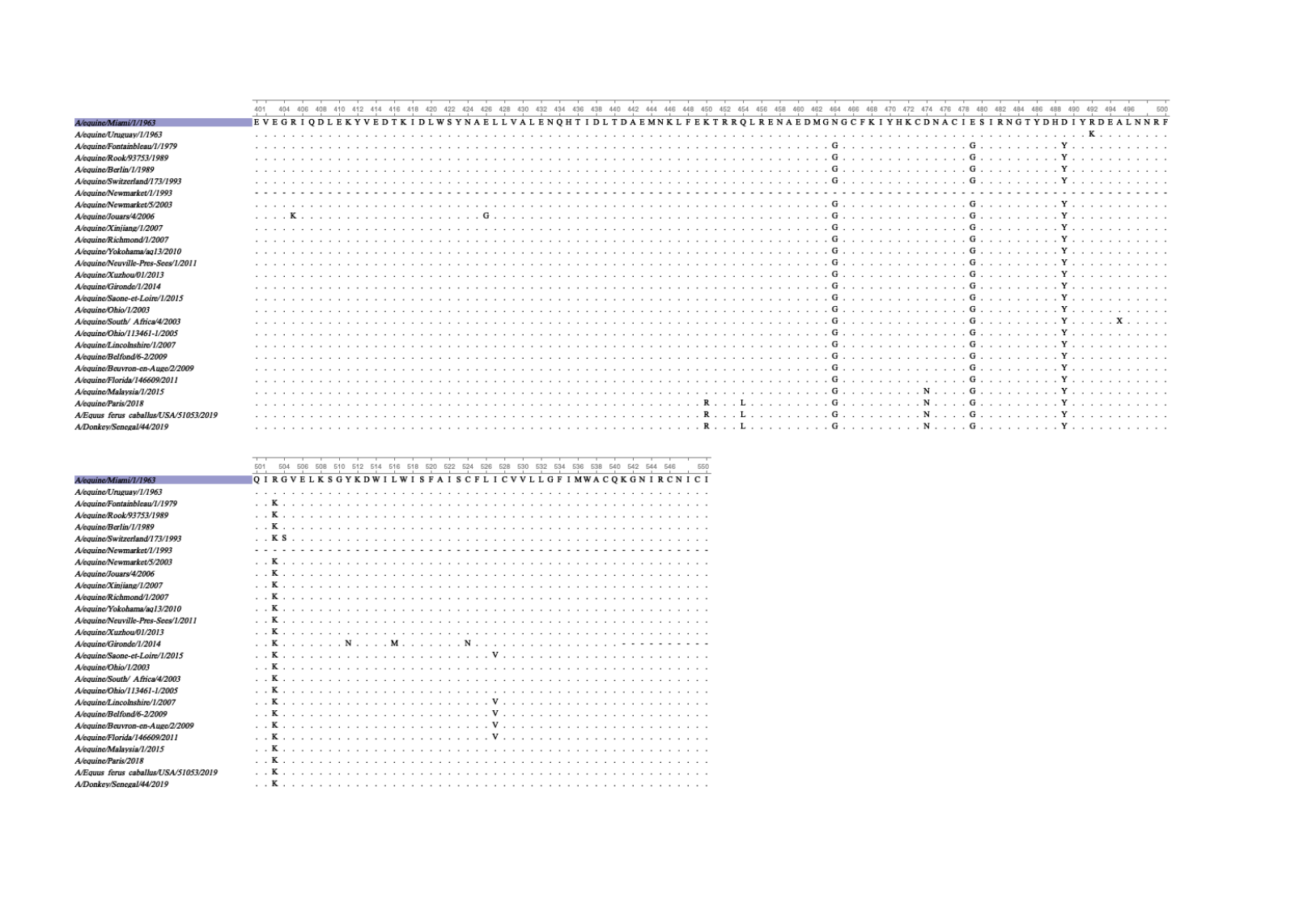

Supplement: Supplementary file 5 — Additional file 5. Multiple alignment of HA amino acid sequences for selected strains since 1963. Antigenic sites are indicated in blue outlined boxes. Amino acid identity is represented with a dot. Absent amino acids are represented with a line. Blue letters (A-E) indicate the antigenic sites. The 130-loop, 190-helix, and 220-loop involved in the receptor-binding site are indicated in orange outlined boxes. [file 13567_2024_1289_MOESM5_ESM.pptx]

## Slide 1
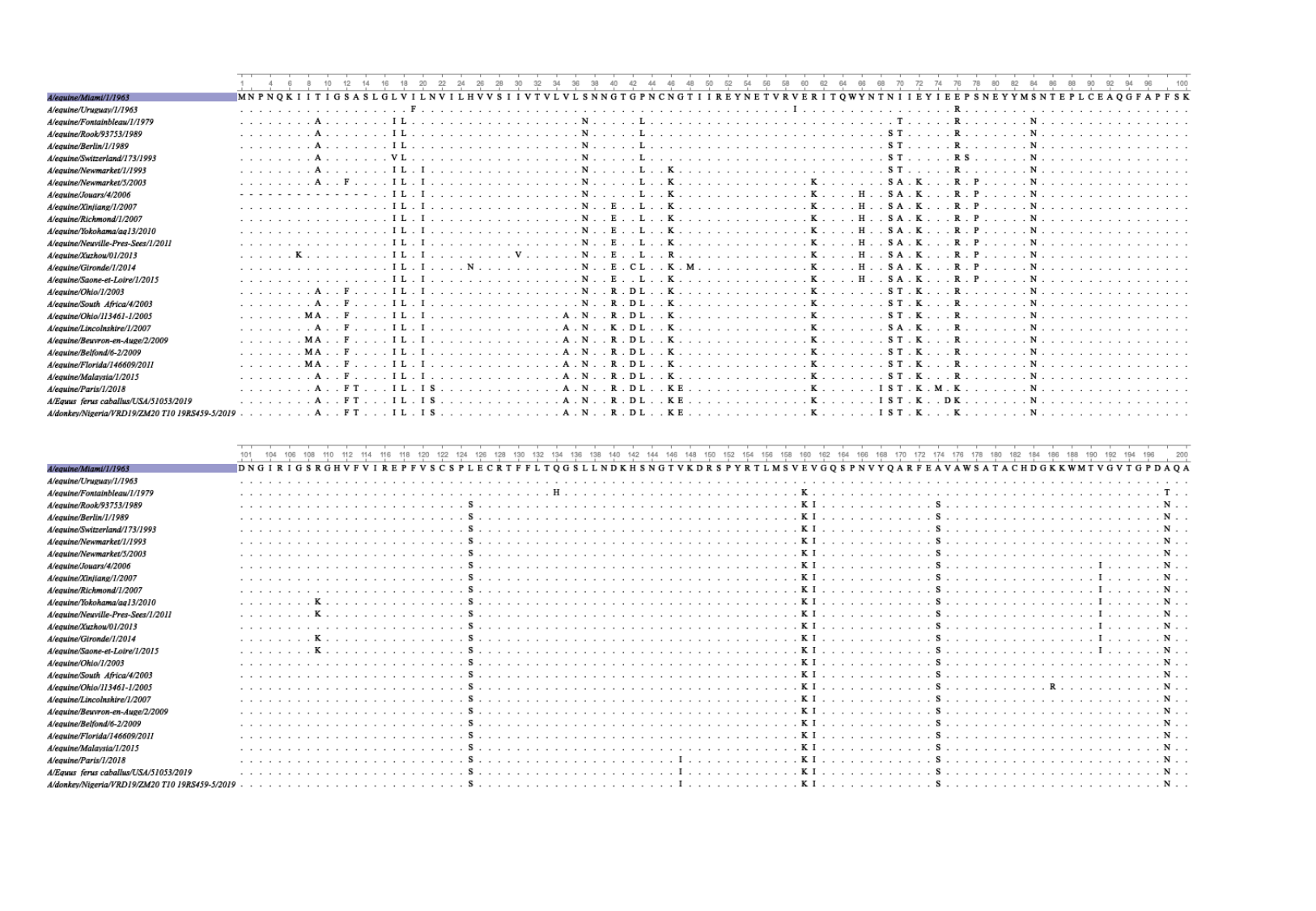

## Slide 2
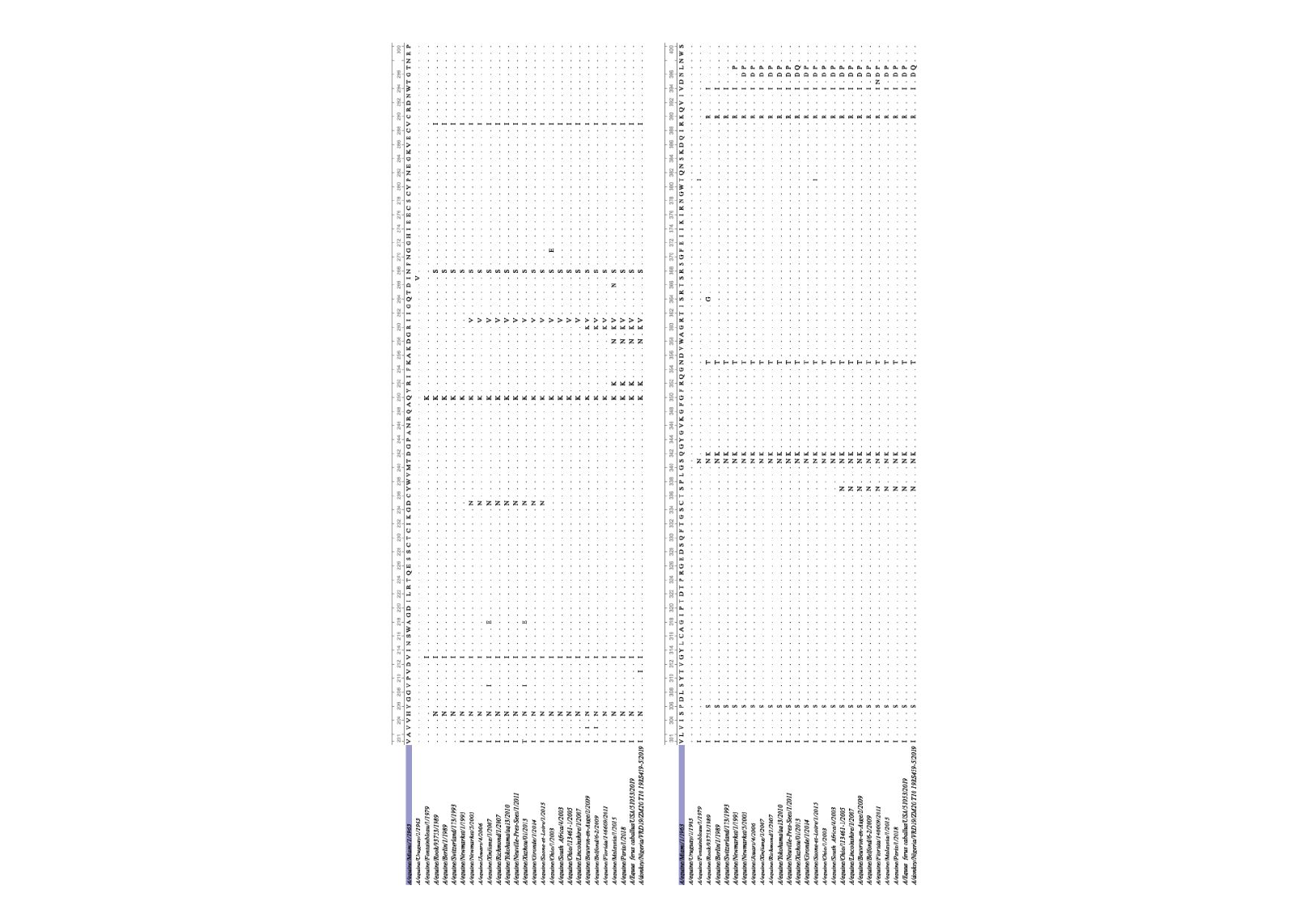

## Slide 3
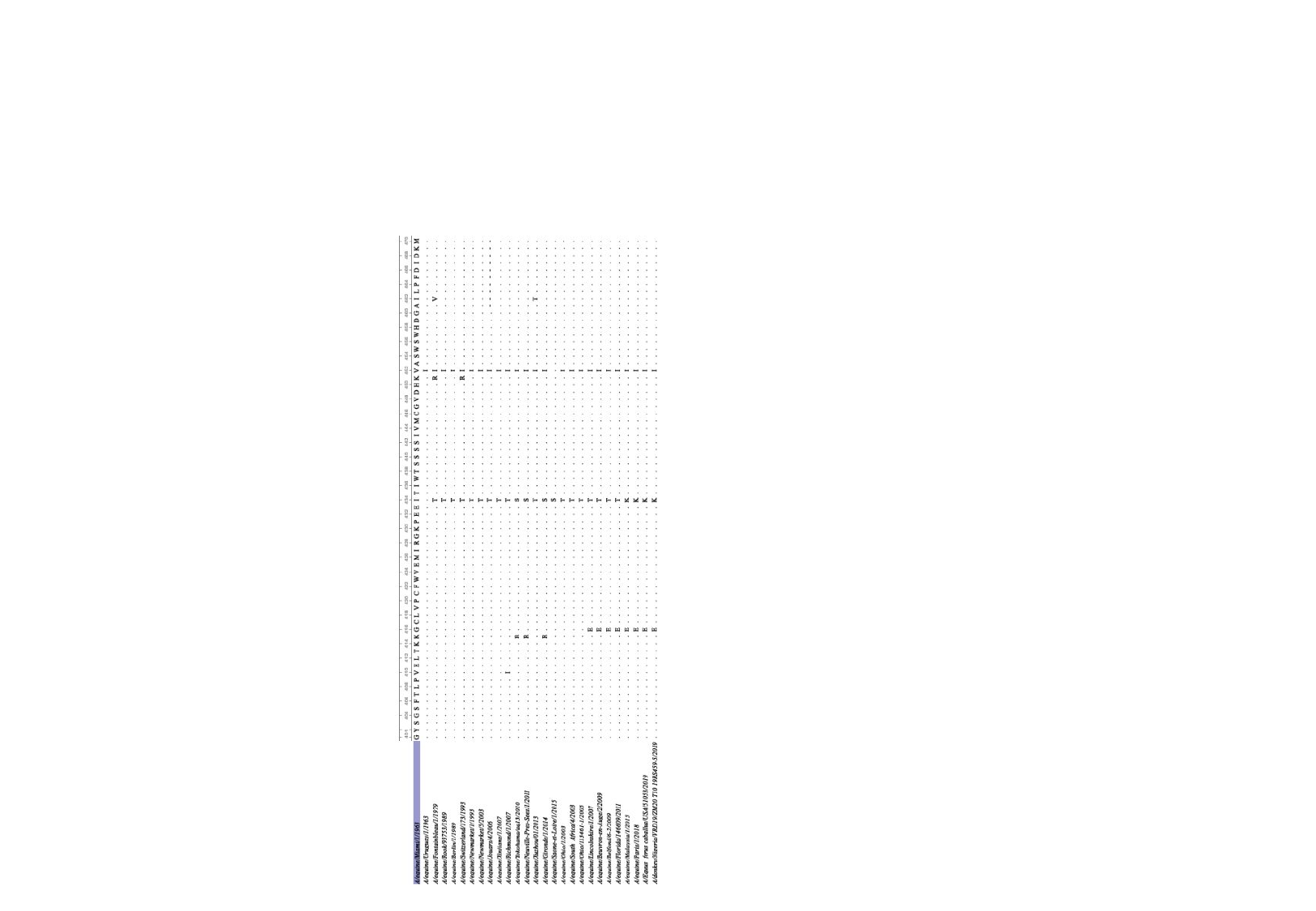

Supplement: Supplementary file 7 — Additional file 7. Multiple alignment of NA amino acid sequences for selected strains since 1963. Amino acid identity is represented with a dot. Absent amino acids are represented with a line. [file 13567_2024_1289_MOESM7_ESM.pptx]
