# Supplementary material for: Genomic characterization of equine influenza A subtype H3N8 viruses by long read sequencing and functional analyses of the PB1-F2 virulence factor of A/equine/Paris/1/2018
Source: Vet Res. 2024 Mar 22;55:36. doi: 10.1186/s13567-024-01289-8 (PMC10960481; doi:10.1186/s13567-024-01289-8)
Supplement: Supplementary file 6 — Additional file 6. Substitutions found in HA and NA. Comparison to A/equine/Ohio/1/2003 for 2009 and 2018 French strains, strain used for MinION consensus sequence, OIE recommended vaccine strains A/equine/South Africa/4/2003 (Fc1) and A/equine/Richmond/1/2007 (Fc2). Numbering according to mature HA. Lines represent identity to A/equine/Ohio/1/2003. [file 13567_2024_1289_MOESM6_ESM.pptx]

## Slide 1
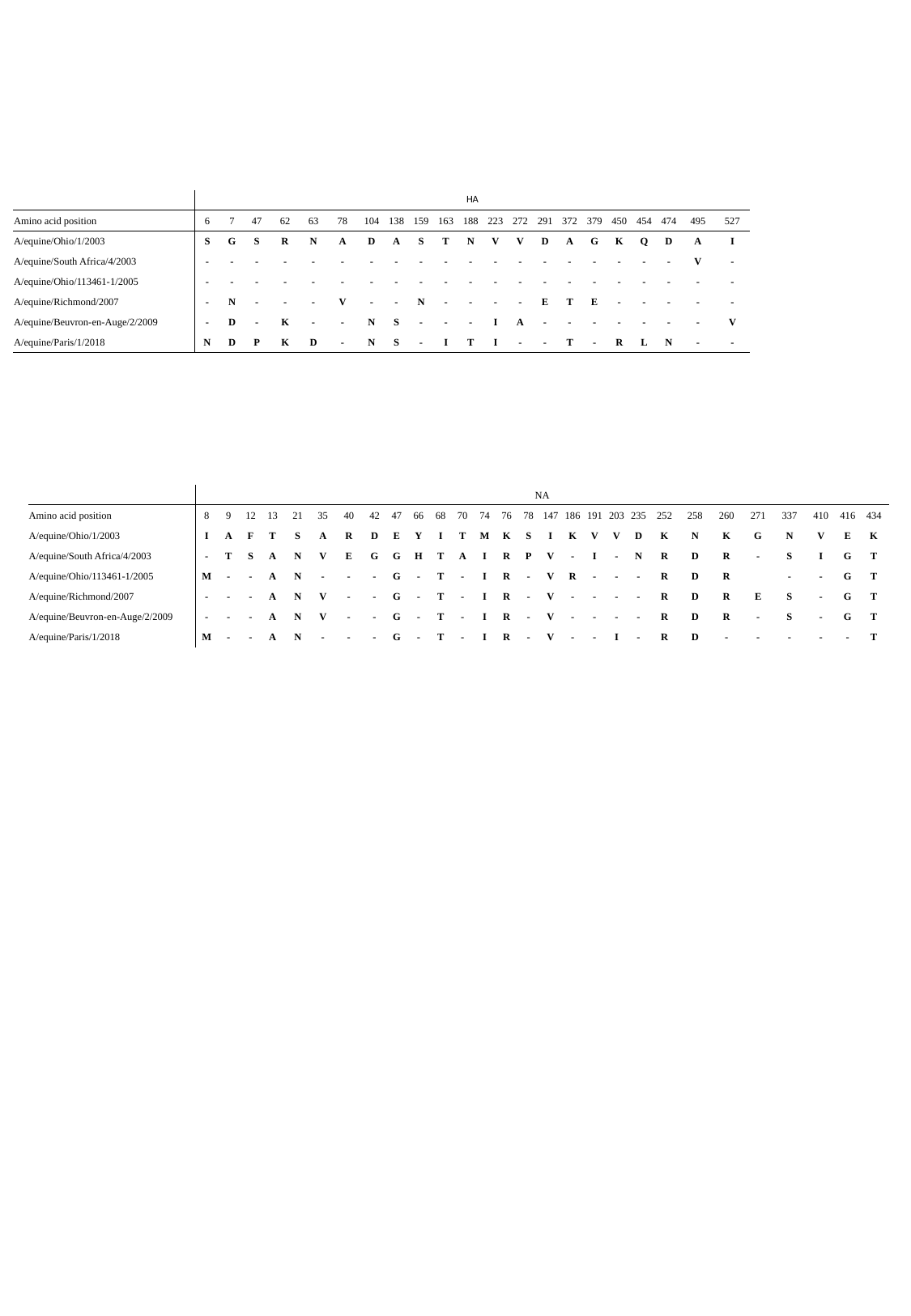

| | HA | | | | | | | | | | | | | | | | | | | | |
| --- | --- | --- | --- | --- | --- | --- | --- | --- | --- | --- | --- | --- | --- | --- | --- | --- | --- | --- | --- | --- | --- |
| Amino acid position | 6 | 7 | 47 | 62 | 63 | 78 | 104 | 138 | 159 | 163 | 188 | 223 | 272 | 291 | 372 | 379 | 450 | 454 | 474 | 495 | 527 |
| A/equine/Ohio/1/2003 | S | G | S | R | N | A | D | A | S | T | N | V | V | D | A | G | K | Q | D | A | I |
| A/equine/South Africa/4/2003 | - | - | - | - | - | - | - | - | - | - | - | - | - | - | - | - | - | - | - | V | - |
| A/equine/Ohio/113461-1/2005 | - | - | - | - | - | - | - | - | - | - | - | - | - | - | - | - | - | - | - | - | - |
| A/equine/Richmond/2007 | - | N | - | - | - | V | - | - | N | - | - | - | - | E | T | E | - | - | - | - | - |
| A/equine/Beuvron-en-Auge/2/2009 | - | D | - | K | - | - | N | S | - | - | - | I | A | - | - | - | - | - | - | - | V |
| A/equine/Paris/1/2018 | N | D | P | K | D | - | N | S | - | I | T | I | - | - | T | - | R | L | N | - | - |
| | NA | NA | | | | | | | | | | | | | | | | | | | | | | | | | | |
| --- | --- | --- | --- | --- | --- | --- | --- | --- | --- | --- | --- | --- | --- | --- | --- | --- | --- | --- | --- | --- | --- | --- | --- | --- | --- | --- | --- | --- |
| Amino acid position | 8 | 9 | 12 | 13 | 21 | 35 | 40 | 42 | 47 | 66 | 68 | 70 | 74 | 76 | 78 | 147 | 186 | 191 | 203 | 235 | 252 | 258 | 260 | 271 | 337 | 410 | 416 | 434 |
| A/equine/Ohio/1/2003 | I | A | F | T | S | A | R | D | E | Y | I | T | M | K | S | I | K | V | V | D | K | N | K | G | N | V | E | K |
| A/equine/South Africa/4/2003 | - | T | S | A | N | V | E | G | G | H | T | A | I | R | P | V | - | I | - | N | R | D | R | - | S | I | G | T |
| A/equine/Ohio/113461-1/2005 | M | - | - | A | N | - | - | - | G | - | T | - | I | R | - | V | R | - | - | - | R | D | R | | - | - | G | T |
| A/equine/Richmond/2007 | - | - | - | A | N | V | - | - | G | - | T | - | I | R | - | V | - | - | - | - | R | D | R | E | S | - | G | T |
| A/equine/Beuvron-en-Auge/2/2009 | - | - | - | A | N | V | - | - | G | - | T | - | I | R | - | V | - | - | - | - | R | D | R | - | S | - | G | T |
| A/equine/Paris/1/2018 | M | - | - | A | N | - | - | - | G | - | T | - | I | R | - | V | - | - | I | - | R | D | - | - | - | - | - | T |
